# Central versus peripheral mechanisms of cold-induced vasodilation: a study in the fingers and toes of people with paraplegia

**DOI:** 10.1007/s00421-023-05175-7

**Published:** 2023-04-02

**Authors:** Lydia Tsoutsoubi, Leonidas G. Ioannou, Billie K. Alba, Stephen S. Cheung, Hein A. Daanen, Igor B. Mekjavic, Andreas D. Flouris

**Affiliations:** 1 FAME Laboratory, Department of Physical Education and Sport Science, University of Thessaly, Karies, 42100 Trikala, Greece; 2 Thermal and Mountain Medicine Division, U.S. Army Research Institute of Environmental Medicine, Natick, MA 01760 USA; 3 Department of Kinesiology, Brock University, St. Catharines, ON Canada; 4 Faculty of Behavioural and Movement Sciences, Vrije Universiteit Amsterdam, Amsterdam, The Netherlands; 5 Department of Automation, Biocybernetics and Robotics, Józef Stefan Institute, 1000 Ljubljana, Slovenia; 6 Department of Biomedical Physiology and Kinesiology, Simon Fraser University, Burnaby, BC V5A 1S6 Canada

**Keywords:** Paraplegia, Water immersion, Sweat rate, Core temperature, Skin temperature

## Abstract

**Purpose:**

This study examined physiological and perceptual parameters related to cold-induced vasodilation (CIVD) in the fingers and toes of people with paraplegia and compared them with responses observed in able-bodied individuals.

**Methods:**

Seven participants with paraplegia and seven able-bodied individuals participated in a randomized matched-controlled study involving left-hand and -foot immersion in cold water (8 ± 1 °C) for 40 min during exposure to cool (16 ± 1 °C), thermoneutral (23 ± 1 °C), and hot (34 ± 1 °C) ambient conditions.

**Results:**

Similar CIVD occurrence was observed in the fingers in the two groups. In toes, three of the seven participants with paraplegia revealed CIVDs: one in cool, two in thermoneutral, and three in hot conditions. No able-bodied participants revealed CIVDs in cool and thermoneutral conditions, while four revealed CIVDs in hot conditions. The toe CIVDs of paraplegic participants were counterintuitive in several respects: they were more frequent in cool and thermoneutral conditions (compared to the able-bodied participants), emerged in these conditions despite lower core and skin temperatures of these participants, and were evident only in cases of thoracic level lesions (instead of lesions at lower spinal levels).

**Conclusion:**

Our findings demonstrated considerable inter-individual variability in CIVD responses in both the paraplegic and able-bodied groups. While we observed vasodilatory responses in the toes of participants with paraplegia that technically fulfilled the criteria for CIVD, it is unlikely that they reflect the CIVD phenomenon observed in able-bodied individuals. Taken together, our findings favor the contribution of central over peripheral factors in relation to the origin and/or control of CIVD.

**Supplementary Information:**

The online version contains supplementary material available at 10.1007/s00421-023-05175-7.

## Introduction

Exposure to a cold stimulus causes vasoconstriction of the cutaneous vasculature to reduce heat loss (Tyler et al. 2020; Van der Struijs et al. 2008; Flouris and Cheung 2010). Paradoxically, during extreme cold exposures, Lewis (1930) reported that this vasoconstrictor response may be interrupted by periods of vasodilation, a phenomenon termed “cold-induced vasodilation” (CIVD). Since its discovery, studies have focussed on the teleological nature of this response, and on the physiological mechanisms involved in its initiation and maintenance. Despite the multitude of studies conducted to address these issues, they remain largely unresolved.

It has been proposed that the purpose of the CIVD response may be cryoprotective, that is to minimize the risk of cold injury (Daanen and Ducharme 1999; Daanen 2003; Cheung and Mekjavic 2007; Cheung 2015; Castellani 2005; Brandstrom et al. 2008; Daanen and van der Struijs 2005). Non-freezing cold injury may result from exposure (typically of the feet) to a cold and wet environment, causing tissue temperatures to be maintained below 15 °C for a prolonged period. In contrast, freezing cold injury may occur when local tissue temperatures decrease below 0 °C (Imray and Oakley 2005; Ingram and Raymond 2013). It is suggested that the periodic reperfusion of the fingers and toes in a scenario of CIVD may minimize, if not prevent, the above-mentioned injury types. For the CIVD response to have a significant cryoprotective effect, the reperfusion of the digits must be such, that it substantially increases the tissue temperature, reflected in higher finger skin temperature (*T*_f_). To date, CIVD has been investigated during local immersion in water temperatures between 0 and 15 °C (Daanen 2003; Mekjavic et al. 2013; Tyler et al. 2015), as well as whole-body or local exposure to air temperatures of − 20 to + 10 °C (Flouris and Cheung 2009b; Flouris et al. 2008; Kramer 1948; Van der Struijs et al. 2008). Also, studies have shown that increased body heat content leads to more frequent and pronounced CIVD reactions, by examining the occurrence and characteristics of *T*_f_ fluctuations during hypothermia, normothermia, and hyperthermia (Daanen and Ducharme 1999; Daanen et al. 1997; Flouris and Cheung 2009b; Flouris et al. 2008).

The CIVD response is characterized primarily by the magnitude of the skin temperature (*T*_sk_) increase, and the duration of this elevated temperature. Studies investigating the CIVD responses have suggested that it should result in a 1 °C of continuous increase in *T*_f_ (Cheung and Mekjavic 2007; Daanen et al. 2012) and be a minimum of 3 min in duration (Mekjavic et al. 2013). Whereas these limits are helpful to standardize the method of investigating the mechanism of CIVD, a response of this magnitude would contribute little to the prevention of a cold injury. Responses that would significantly impact the prevention of cold injury have been reported (i.e., ∆*T*_sk_ > 10 °C), but are more an exception than a rule. Understanding the origin of the CIVD response is not only of interest from a mechanistic perspective but also may allow strategies to be developed that would harness an existing response to provide cold injury prevention. Namely, training strategies that would enhance the response, such as repeated foot immersions for 15 days before the exposure that can lead to small increases in toe temperature (Daanen et al. 2012), or endurance training programs that can improve mean finger temperature (Keramidas et al. 2010). In this regard, one of the issues that requires resolution is whether the CIVD response is purely a local reaction, whether it is governed by central mechanisms, or a combination of the two.

Accordingly, the aim of this study was to examine physiological and perceptual parameters related to CIVD in the fingers and toes of people with paraplegia and compare them with responses observed in able-bodied individuals during exposure to cool, thermoneutral, and hot environments. We reasoned that a predominantly neurally mediated CIVD response would rely on the thermal afferent information emanating from the cutaneous cold sensors and the subsequent centrally mediated thermoeffector drive to the peripheral cutaneous vasculature. A lesion in the spinal cord above the level at which afferent and efferent nerves for a given region enter and exit the spinal cord, respectively, would abolish the neural traffic (Price and Trbovich 2018; Nicotra et al. 2004; Nash et al. 1996), thus eliminating the likelihood of a CIVD response, if the response were solely neural in origin. In contrast, the rapid conductive cooling of the digits could initiate local mechanisms, such as either a direct thermal effect on the cutaneous vasculature or an indirect humoral effect. This would be reflected in a similar CIVD response of paraplegic and able-bodied participants. It is also possible that both mechanisms are involved in the CIVD response, with perhaps one predominating over the other. Although the relative contribution of each of these mechanisms in able-bodied individuals to CIVD is unknown, it is most likely that with the abolition of the neural mechanism in paraplegic individuals, any CIVD response would probably be of a local origin. Spinal cord injuries are not similar, even if they are at the same level. Based on the severity (complete or incomplete spinal cord injury), some paraplegics may have vestiges of innervation below the lesion (Krassioukov and West 2014). This would be apparent in the whole-body perceptual responses.

Accordingly, in this study, we examined physiological and perceptual parameters during cold water immersions in people with paraplegia and able-bodied individuals with the aim to investigate the origin of the CIVD response, namely whether it is central or peripheral. Under the assumption that paraplegic participants have no central control over blood vessels under the level of lesion, the CIVD reactions in paraplegic and able-bodied participants were investigated during exposure to different environmental conditions, resulting in different levels of heat content, since body heat content is known to significantly influence CIVD reactions (Daanen and Ducharme 1999; Daanen et al. 1997; Flouris and Cheung 2009b; Flouris et al. 2008). In addition to the vasomotor responses of the fingers and toes, sudomotor responses (i.e., sweating) in the present study were also monitored at the forehead and calf. The hypothesis being that particularly during the hot exposure, differences in the sweating response of regions innervated by nerves above and below the spinal cord lesion (Handrakis et al. 2017; Karlsson et al. 2012; Price and Trbovich 2018) would provide further support of impairment of a thermoregulatory effector in regions below the lesion.

## Materials and methods

### Ethical approval and participants

The experimental protocol (ClinicalTrials.gov ID: NCT04215939) conformed to the standards set by the Declaration of Helsinki and was approved by the Bioethics Review Board of the University of Thessaly Department of Physical Education and Sport Science (protocol no.: 1320). Participants were requested to provide a medical history to exclude patients with Raynaud’s syndrome/phenomenon as well as those under prescription medication for hypertension or other drugs that could affect vasomotion. Three out of 14 participants (one able-bodied and two paraplegic participants) were smokers and were asked to refrain from smoking at least 10 h prior to each experimental session. Physiological data as well as the number of CIVDs from the six males and one female able-bodied participants have been previously published to investigate the cardiovascular stress and the characteristics of CIVD in women and men during cold water immersion (Tsoutsoubi et al. 2022). All volunteers were given a full explanation of all the procedures and signed a written informed consent prior to participating in the study.

**Fig. 1:**
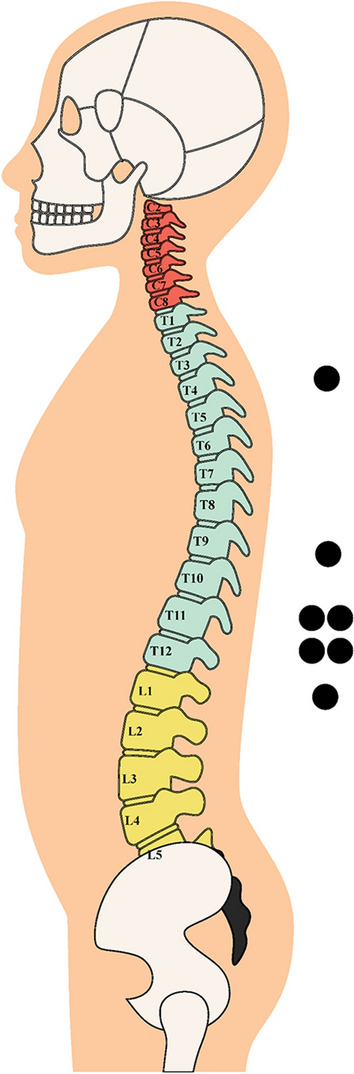
Level of injury for participants with paraplegia

The minimum required sample size for investigating “repeated-measures, within–between factors” was calculated using the results of a previous repeated-measures study (Daanen and Ducharme 1999) which assessed CIVDs in able-bodied individuals who were in thermoneutral, mildly hypothermic, and mildly hyperthermic conditions. Specifically, we calculated the effect size (*f*) (Cohen 1988) for the comparisons reported in the previously published study regarding minimum and maximum *T*_f_ among fingers as well as among participants (Daanen and Ducharme 1999). To ensure high statistical power, our calculation of the minimum required sample size was based on the lowest effect size identified. This was an effect size (*f*) equal to 0.5 [equating to an effect size (*d*) of 1.0] for the comparison of maximum *T*_f_ in the mild hypothermic (8.3 ± 1.7 °C) versus the thermoneutral (11 ± 3.2 °C) condition (Daanen and Ducharme 1999). Sample size calculations were conducted using G*Power 3.1.9.4 (Faul et al. 2007), while setting statistical power and α error probabilities at 0.90 and 0.05, respectively. Based on this process, six participants per group (12 participant in total) would provide sufficient power to detect differences between able-bodied and paraplegic individuals in our study. Therefore, six males and one female with paraplegia as well as an equivalent number of able-bodied males and female matched for age, body mass index, and body surface area (i.e., *p* > 0.05 between groups) participated in the study. The anthropometric characteristics of all 14 participants are shown in Table 1. The average time since injury of those with paraplegia was 13 ± 15 years (range 2–40 years), and all had complete motor paralysis of the legs with a diagnosis of ASIA A, following a clinical neurological exam which was registered in the public healthcare system. Of the paraplegic participants, one had suffered an injury in the T4, one in the T9, two in the T11, two in the T12, and one in the L1 (Fig. 1). Therefore, no neurological changes would be expected in the fingers of the paraplegic participants as the injury sites were below innervation to the hands/fingers.

**Table 1:** Anthropometric characteristics of the participants in the two groups

Group	*n*	Age (years)	Body fat (%)	Weight (kg)	Height (m)	BMI (kg/m^2^)	BSA (m^2^)
Paraplegic	7	34.1 ± 11.3	39.5 ± 11.5	73.0 ± 15.2	1.78 ± 0.1	23.5 ± 6.4	1.9 ± 0.2
Able-bodied	7	35.0 ± 11.4	28.7 ± 7.6	80.7 ± 18.3	1.76 ± 0.1	25.9 ± 5.2	2.0 ± 0.2

*BMI* body mass index, *BSA* body surface area

### Experimental protocol

The study included a familiarization session and three experimental sessions. During the familiarization session, participants were informed about all data collection procedures/equipment and underwent anthropometric and body composition (Dual-energy X-ray absorptiometry; (Lunar model DPX Madison, WI).) assessments. Thereafter, participants were requested to undergo three trials, in each being exposed to different environmental conditions inside a 32.5 m^3^ environmental chamber (HGX22G/190-4, ABB, Germany): cool environment (wet-bulb globe temperature: 10.8 °C; air temperature: 16 ± 1 °C; relative humidity: 45 ± 5%; air velocity: 0.2 m/s; solar radiation: 0 W/m^2^), neutral (wet-bulb globe temperature: 17.2 °C; air temperature: 23 ± 1 °C; relative humidity: 45 ± 5%; air velocity: 0.2 m/s; solar radiation: 0 W/m^2^), and hot (wet-bulb globe temperature: 27.2 °C; air temperature: 34 ± 1 °C; 45 ± 5% relative humidity; air velocity: 0.2 m/s; solar radiation: 0 W/m^2^) environments. The overall thermal stress experienced by the participants in our study was expressed by means of wet-bulb globe temperature which was found to be the most efficacious thermal stress indicator for assessing the physiological strain (Ioannou et al. 2022a, b, c). The experimental sessions were administered in a random order, based on a random allocation algorithm implemented in Excel Spreadsheets (Microsoft Office, Microsoft, Washington, USA).

**Fig. 2:**
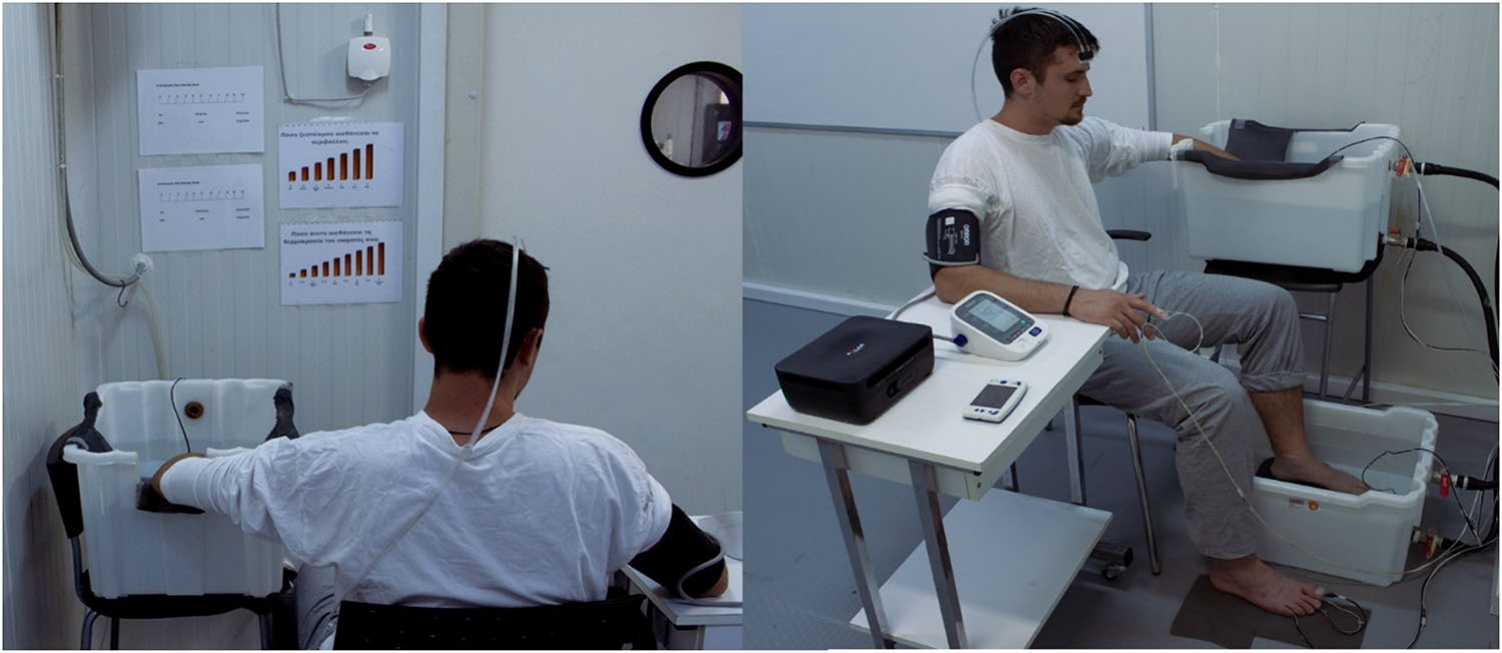
An able-bodied participant during the data collection. The right sleeve of the shirt was cut at elbow height to avoid constricting blood flow. The blood pressure cuff was comfortably placed at the upper arm without constricting blood flow except when assessing arterial blood pressure

For each session, participants arrived at the same time of the day. They were requested to refrain from caffeine for at least two hours, from food for at least three hours, and from alcohol and exercise for at least 12 h prior to experiment. Upon arrival, participants dressed down to a long-sleeve shirt and a pair of pants 100% cotton (Fig. 2). The room temperature in the lab where the participants changed clothes before entering the environmental chamber was thermoneutral (wet-bulb globe temperature: 16.3 °C; air temperature: 22 °C; relative humidity: 45%; air velocity: 0.2 m/s; solar radiation: 0 W/m^2^). The time spent in this environment (for dressing down and to ask any further questions) was about 10–15 min. The estimated clothing insulation was 0.67 clo for males (underwear 0.04; long-sleeve shirt 0.29; pants: 0.34) and 0.69 clo for females (underwear 0.04; bra: 0.02; long-sleeve shirt 0.29; pants: 0.34). (American Society of Heating 2004; Ioannou et al. 2019). Thereafter, participants entered the environmental chamber and instrumentation took place for 10–15 min. Once all sensors were applied, participants were requested to relax seated for 20 min with their hands supported at the level of the heart (Fig. 2). After the baseline period, participants were asked to simultaneously immerse their left hand (up to the ulnar styloid process) and left foot (up to the lateral malleolus) in warm water (35 ± 1 °C) for five minutes to ensure similar starting local tissue temperature (Daanen et al. 2012). Thereafter, the left hand and foot were immersed in cold water (8 ± 1 °C) for 40 min (Fig. 2). After the end of the cold water immersion period, the left hand and foot were removed from the water and participants remained seated for an additional five minutes to monitor the recovery phase. The total duration of the experiment was 70 min: 20-min baseline period, 5-min warm water immersion, 40-min cold water immersion, and 5-min recovery.

The water tanks used for the warm immersion were 0.58 m^3^ for the hand and 0.85 m^3^ for the foot. The water tanks used for the cold immersion were 0.91 m^3^. Water temperature was controlled within 1 °C using an air-to-water heat pump (model KF120-B, Dongguan, China) which was adjusted to continuously circulate the water to ensure uniform exposure. During the water immersion periods, the left foot was placed in a water tank on the floor, and the right foot rested on the floor (Fig. 2). Water-permeable mesh fabrics were used to line the bottom of the water tanks and the floor to ensure that the immersed hand and foot made no contact with the bottom of the water tanks, and that the non-immersed hand and foot made no contact with the floor of the environmental chamber (Fig. 2).

### Measurements

**Fig. 3:**
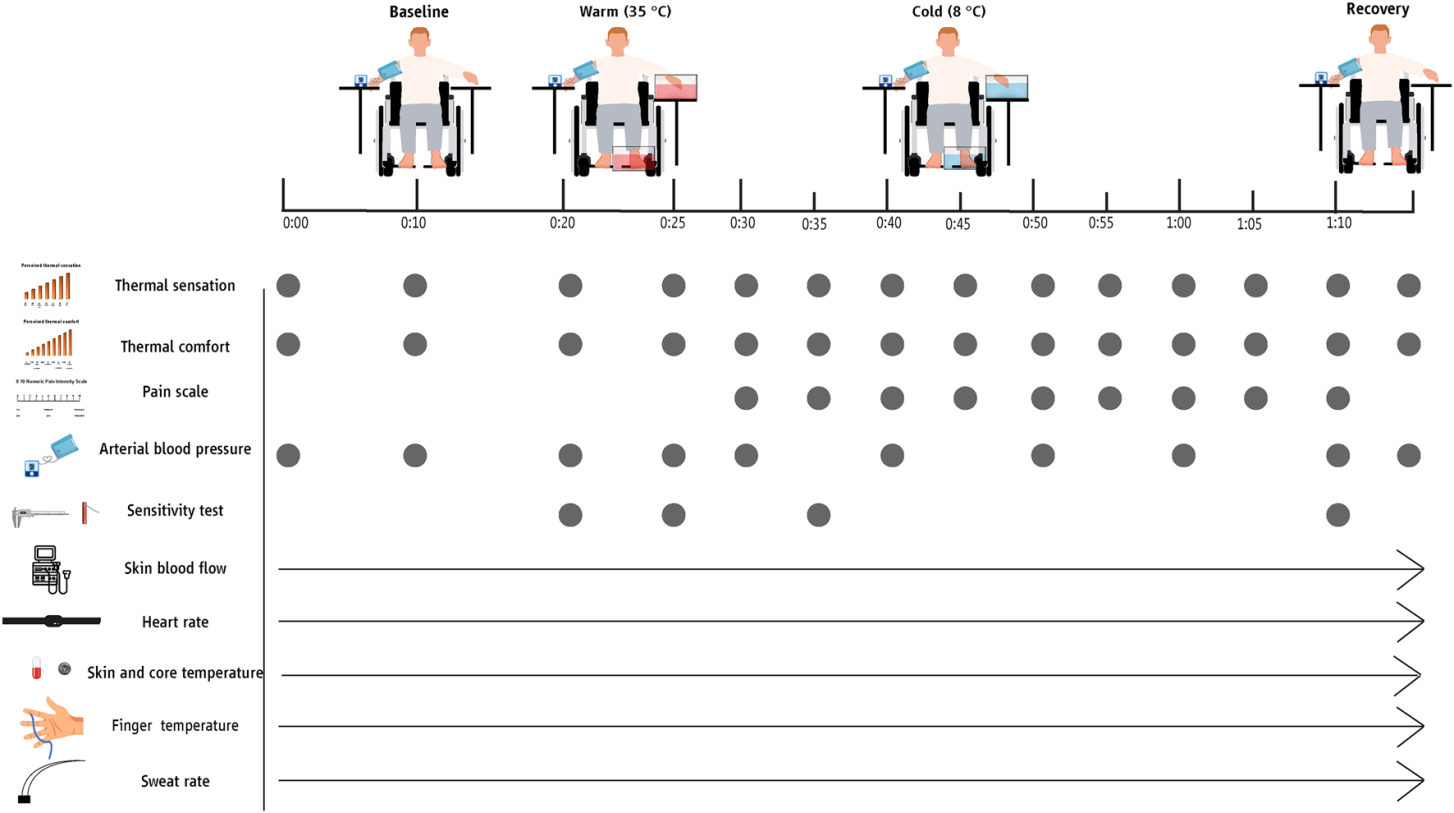
Physiological and perceptual measurements during the experimental protocol

Gastro-intestinal (*T*_gi_) and mean skin (*T*_sk_) temperature (both assessed in 1-min intervals), as well as finger temperature (*T*_f_), toe temperature (*T*_t_), skin blood flow (SkBF), sweat rate, and heart rate were continuously monitored. Raw data from these variables—assessed in 0.05 s (for SkBF), 1 s (for heart rate), 8 s (for *T*_f_ and *T*_t_), and 10 s (for sweat rate) intervals—were used to provide 1-min averages which were used for all statistical analyses. In addition, 20-s averages were calculated for *T*_f_, *T*_t_, and SkBF and were used to identify CIVD reactions (Fig. 3). In terms of the remaining measurements, arterial blood pressure was assessed on the arm of the non-immersed hand every 10 min during baseline, at the end of the warm immersion, every 10 min during cold water immersion, and at the end of the recovery period (Fig. 3). Thermal comfort and thermal sensation were recorded every 10 min during baseline and every 5 min during cold water immersion. Pain sensation was assessed at the start of cold immersion and then every five minutes (Fig. 3). Tactile sensitivity of the immersed limbs was tested at the end of the baseline period, at the end of the warm water immersion, as well as at minutes 5 and 40 of the cold water immersion (for the latter two, the hand and foot were briefly removed from the water) (Fig. 3).

Gastro-intestinal temperature (*T*_gi_) was monitored using telemetric capsules (BodyCap, Caen, France) as an indicator of gastrointestinal (core) temperature. Although the time following ingestion does not significantly influence the validity of telemetry capsule measurements of core temperature in the absence of fluid consumption (Notley et al. 2021), the capsules were always ingested at the same time before the start of the protocol for each participant. Skin temperature from four sites (arm, chest, leg and thigh) was recorded using wireless thermistors (iButtons type DS1921H, Maxim/Dallas Semiconductor Corp., USA) and was used to calculate *T*_sk_ according to Ramanathan [*T*_sk_ = 0.3(chest + arm) + 0.2(thigh + leg)] (Ramanathan 1964). Mean body temperature (*T*_b_) was calculated by Burton in 1935: *T*_b_ = 0.64 × *T*_gi_ + 0.36 × *T*_sk_ (Burton 1935).

Using surgical tape (3 M Transpore Tape, 3 M Canada), six ceramic chip skin thermistors (MA-100, Thermometrics) were attached on the lower part of the pad on the 2nd finger (i.e., index finger) of both hands and on the lower part of the pad on the 1st, 3rd, and 5th toes of the immersed foot, as well as at on the 1st toe of the non-immersed foot. Data were recorded using a data logger (Smartreader 8 Plus, ACR, Vancouver, Canada).

Skin blood flow (SkBF) was monitored with a laser Doppler flowmeter (PF4000 LDPM, Perimed, Stockholm, Sweden, PF5010 LDPM, Perimed, Stockholm, Sweden) at the pad of the 2nd finger in each hand as well as at the distal edge of the 1st toe of each foot. The probe (PR 407 small straight probe, Perimed) on the non-immersed 2nd finger was held in place with a plastic mini holder (diameter: 5 mm; PH 07-5, Perimed), which was fixed to the skin using double-sided adhesive strips (PF 105-3, Perimed) without constricting the finger. All other probes (413 Integrating Probe, Perimed, Stockholm, Sweden) were held in place with a plastic holder (PH 13, Perimed, Stockholm, Sweden). The SkBF data were expressed as absolute values in perfusion units (PU).

Sweat rate was measured at the forehead and the belly of the gastrocnemius using the ventilated capsule method (SFM4100, Sensirion, Staefa, Switzerland). Heart rate was monitored using a wireless heart rate system (Polar Team2, Polar Electro Oy, Kempele, Finland). Arterial blood pressure was assessed on the arm of the non-immersed hand (Omron Healthcare, M6 comfort, Kyoto, Japan). Whole body thermal comfort (from 1 = comfortable to 5 = extremely uncomfortable) and thermal sensation (from −3 = cold to + 3 = hot) were recorded using standard scales (Gagge et al. 1967). Pain intensity (from 1 = no pain to 10 = worst pain imaginable), as well as pain distress [in Likert (from 1 = no pain to 10 = unbearable pain) and visual analog scale (no pain to unbearable pain)] were recorded using standardized scales (Daanen et al. 2012). Tactile sensitivity of the tip of the middle finger (for all participants) and of the tip of the second toe (for able-bodied individuals) of the immersed limbs was assessed using Semmes–Weinstein monofilaments and a digital esthesiometer, based on the previous methods (Daanen et al. 2012; Sapa et al. 2019). For both measurements, lower values indicate increased tactile sensitivity.

### Statistical analysis

The 20-s average values for *T*_f_ and *T*_t_ were used to visually identify CIVDs based on previous criteria (Daanen 2003; Daanen et al. 2012) as follows:

1. Number of waves (*N*): The number of each wave that fulfill the criterium of an increase of at least 1 °C.
2. Minimum temperature (*T*_min_): the lowest temperature just before the start of CIVD in °C.
3. Maximum temperature (*T*_max_): the highest temperature during the CIVD in °C.
4. Onset time (*T*_onset_): the time from immersion to *T*_min_ in minutes.
5. Peak time (*T*_peak_): the time to reach the maximum temperature (time at *T*_max_ minus time at *T*_min_) in minutes.
6. Average temperature (*T*_avg_): the average temperature during the cold water immersion period without the first five minutes of the cold immersion in °C.
7. Temperature amplitude (Δ*T*): the difference between *T*_min_ and *T*_max_ in °C.

A Shapiro–Wilks test was used to test the normality assumption in continuous variables, demonstrating that they were distributed normally. Chi-square tests were used to compare the frequency of CIVDs across the three different environments, the fingers/toes, as well as between the hand and the foot. Since the aim of the study was to identify differences between matched groups the mean data for each phase were used to perform paired sample *t*-tests as well as effect sizes (*d*) to detect differences between the participants with paraplegia and able-bodied controls across all the collected variables [*T*_f_, *T*_t_, SkBF, *T*_sk_, *T*_gi_, arm/chest/leg/thigh skin temperature, perceptual scales (thermal comfort, thermal sensation, pain), systolic blood pressure, diastolic blood pressure, heart rate]. The comparisons were performed separately for each phase of the protocol (baseline, warm immersion, cold immersion, recovery). The level of statistical significance in the t-tests was adjusted for multiple comparisons using the Bonferroni correction, resulting in a level of significance of 0.008. The magnitude of Cohen’s (*d*) effect sizes was determined as follows: *d* (0.01) = very small; *d* (0.2) = small; *d* (0.5) = medium; *d* (0.8) = large; *d* (1.2) = very large; and *d* (2.0) = huge (Sawilowsky 2009). Finally, in participants with paraplegia, we also computed correlation coefficients for the relationship between the vertebrae number (with C1 coded as “1” and L5 coded as “25”) and all the collected variables to investigate if the level of injury is linked with the severity of changes in the physiological and perceptual data. Effect sizes (*d*) were computed with Excel Spreadsheets (Microsoft Office, Microsoft, Washington, USA) and all other analyses were conducted with SPSS v27.0 (IBM, Armonk, NY, USA).

## Results

### Frequency of CIVD

According to the above definition of CIVD, we observed 75 CIVDs in both groups, distributed as follows: 13 in the cool environment, 17 in the thermoneutral environment, and 45 in the hot environment (*p* < 0.001). When analyzing data only for the toes, one participant demonstrated CIVD in the cool environment (paraplegic group: 1; able-bodied group: 0; Table 2), two participants demonstrated CIVD in the thermoneutral environment (paraplegic group: 2; able-bodied group: 0; Table 3), and seven participants demonstrated CIVD in the heat (paraplegic group: 3; able-bodied group: 4; Table 4). When considering all participants simultaneously, chi-square analysis demonstrated that a significantly (*p* = 0.008) higher number of participants demonstrated CIVD in the toes during exposure in the heat (*n* = 7) compared to the thermoneutral (*n* = 2; *p* = 0.035) and the cool (*n* = 1; *p* = 0.005) environment. With regards to the fingers, eight participants demonstrated CIVD in the cool environment (paraplegic group: 2; able-bodied group: 6; *p* = 0.031; Table 2), eight in the thermoneutral environment (paraplegic group: 4; able-bodied group: 4; *p* = 1.000; Table 3), and nine in the hot environment (paraplegic group: 5; able-bodied group: 4; *p* = 0.505; Table 4) (*p* > 0.05). When considering all participants simultaneously, chi-square analysis (*p* = 0.631) demonstrated that the number of participants exhibiting CIVD in the fingers was not different during exposure in the heat (*n* = 9) compared to the thermoneutral (*n* = 8; *p* = 0.653) and the cool (*n* = 8; *p* = 0.340) environment.

**Table 2:**
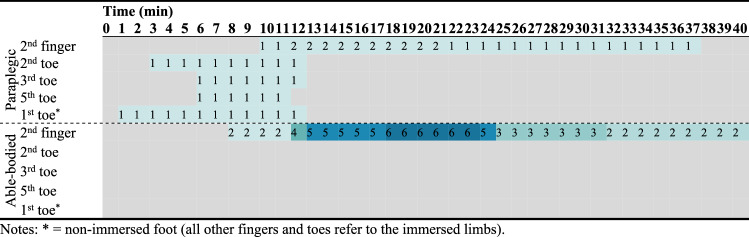
Heat map presenting the number of paraplegic and able-bodied participants that demonstrated a CIVD reaction for each minute of the cold immersion during exposure in the cool environment (16 ± 1 °C)

Colors indicate frequencies. Gray color indicates lack of CIVD. * = non-immersed foot (all other fingers and toes refer to the immersed limbs)

**Table 3:**
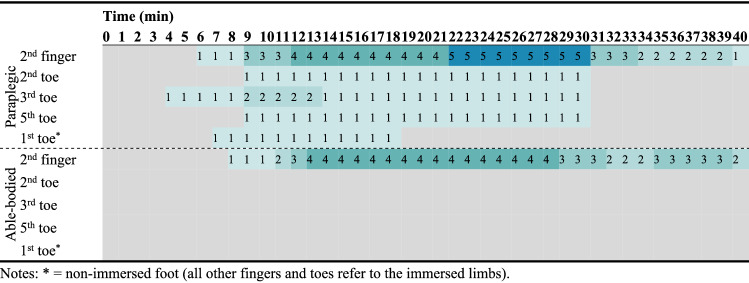
Heat map presenting the number of paraplegic and able-bodied participants that demonstrated a CIVD reaction for each minute of the cold immersion during exposure in the thermoneutral environment (23 ± 1 °C)

Colors indicate frequencies. Gray color indicates lack of CIVD. * = non-immersed foot (all other fingers and toes refer to the immersed limbs)

**Table 4:**
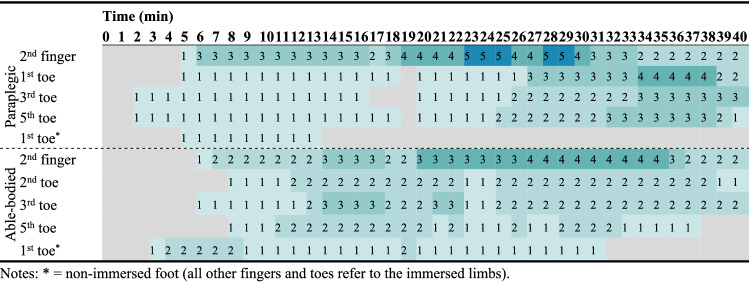
Heat map presenting the number of paraplegic and able-bodied participants that demonstrated a CIVD reaction for each minute of the cold immersion during exposure in the hot environment (34 ± 1 °C)

Colors indicate frequencies. Gray color indicates lack of CIVD. * = non-immersed foot (all other fingers and toes refer to the immersed limbs)

For CIVDs detected in the toes, one participant (level of lesion T4) in the paraplegic group revealed simultaneous and synchronous CIVDs across all toes in the cool environment, two other participants (level of lesion T11 and T12) showed simultaneous and synchronous CIVDs in the thermoneutral environment, and all three participants demonstrated simultaneous and synchronous CIVDs in the hot environment (*p* = 0.383 for the effect of environment on CIVD occurrence). These were the same three participants, while the other four participants in the paraplegic group showed no CIVDs in the toes. In the able-bodied group, none of the participants revealed CIVDs in the toes in the cool and thermoneutral environments, while four participants revealed CIVDs in the heat (*p* = 0.004). Overall, we found significant differences in the number of participants demonstrating CIVDs in the toes across groups and environments (i.e., participants with CIVD in the toes × group × environment; *p* = 0.034), with CIVDs being more prevalent in paraplegic participants and in the heat compared to the thermoneutral and cool environments.

For the CIVDs detected in the fingers, two participants in the paraplegic group revealed CIVDs in the cool environment, four in the thermoneutral environment, and five in the heat (*p* = 0.119). In the able-bodied group, six participants revealed CIVDs in the fingers in the cool environment, four in the thermoneutral environment, and four in the heat (*p* = 0.661). Overall, we found no differences in the number of participants demonstrating CIVDs in the fingers across groups and environments (*p* = 0.272). However, a post hoc chi-square test showed that CIVD in the fingers during exposure to the cool environment was more prevalent in the able-bodied group (*p* = 0.031).

### Characteristics of CIVD

**Fig. 4:**
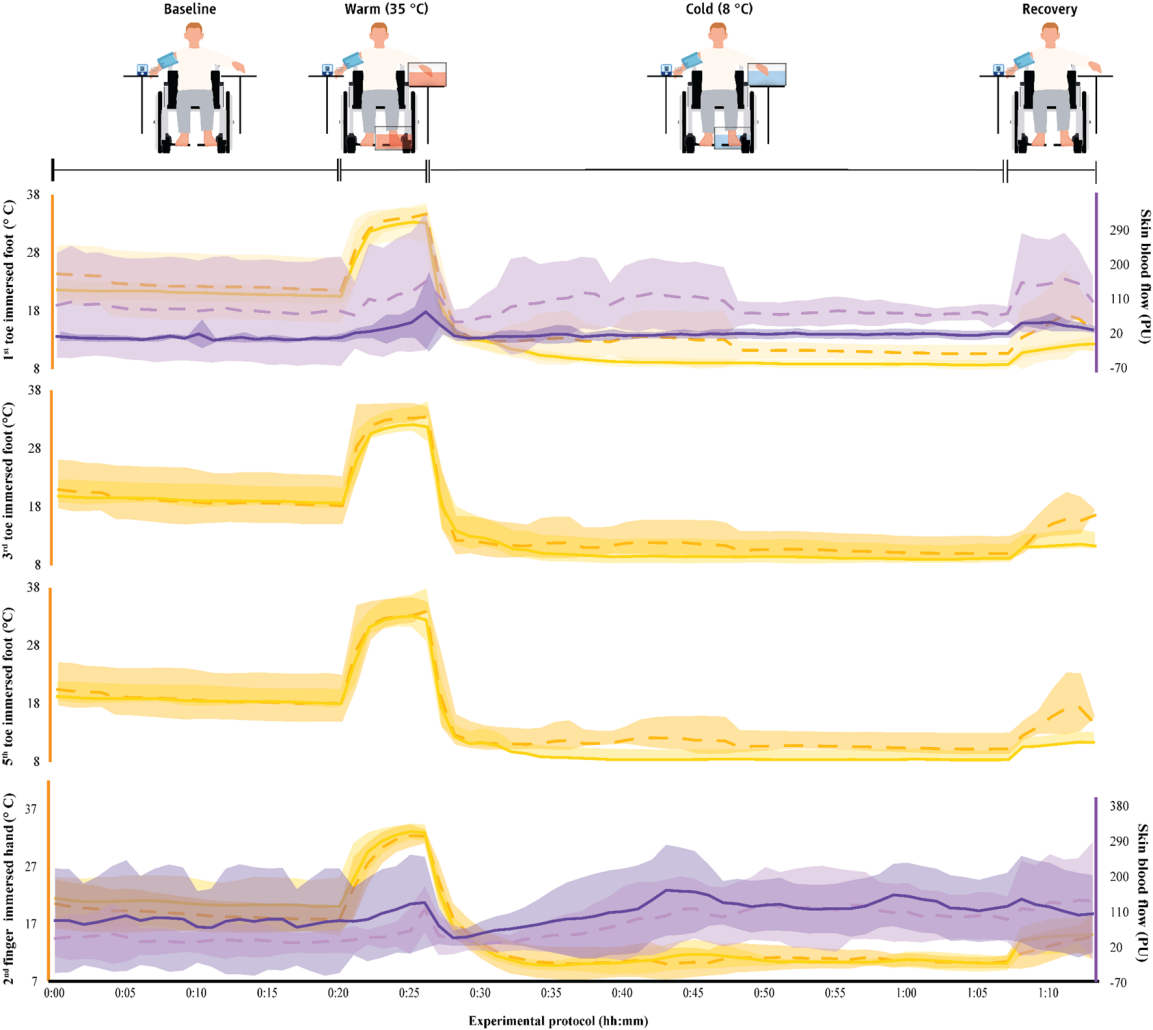
Skin temperature in the fingers and toes as well as skin blood flow (mean ± SD) in the cool environment (16 ± 1 °C) in the two groups. The first 20 min (00:00–00:20) indicate data collected during the baseline phase, the next five min (00:20–00:25) indicate responses during the warm immersion, the next 40 min (00:30–00:70) indicate responses during the cold immersion, and the final five min (00:70–00:75) show responses during the recovery phase. Finger and toe temperatures are indicated with continuous yellow lines in paraplegic participants and with dashed yellow lines in the able-bodied individuals. Finger and toe skin blood flow data are indicated with continuous purple lines in paraplegic participants and with dashed purple lines in able-bodied individuals

**Fig. 5:**
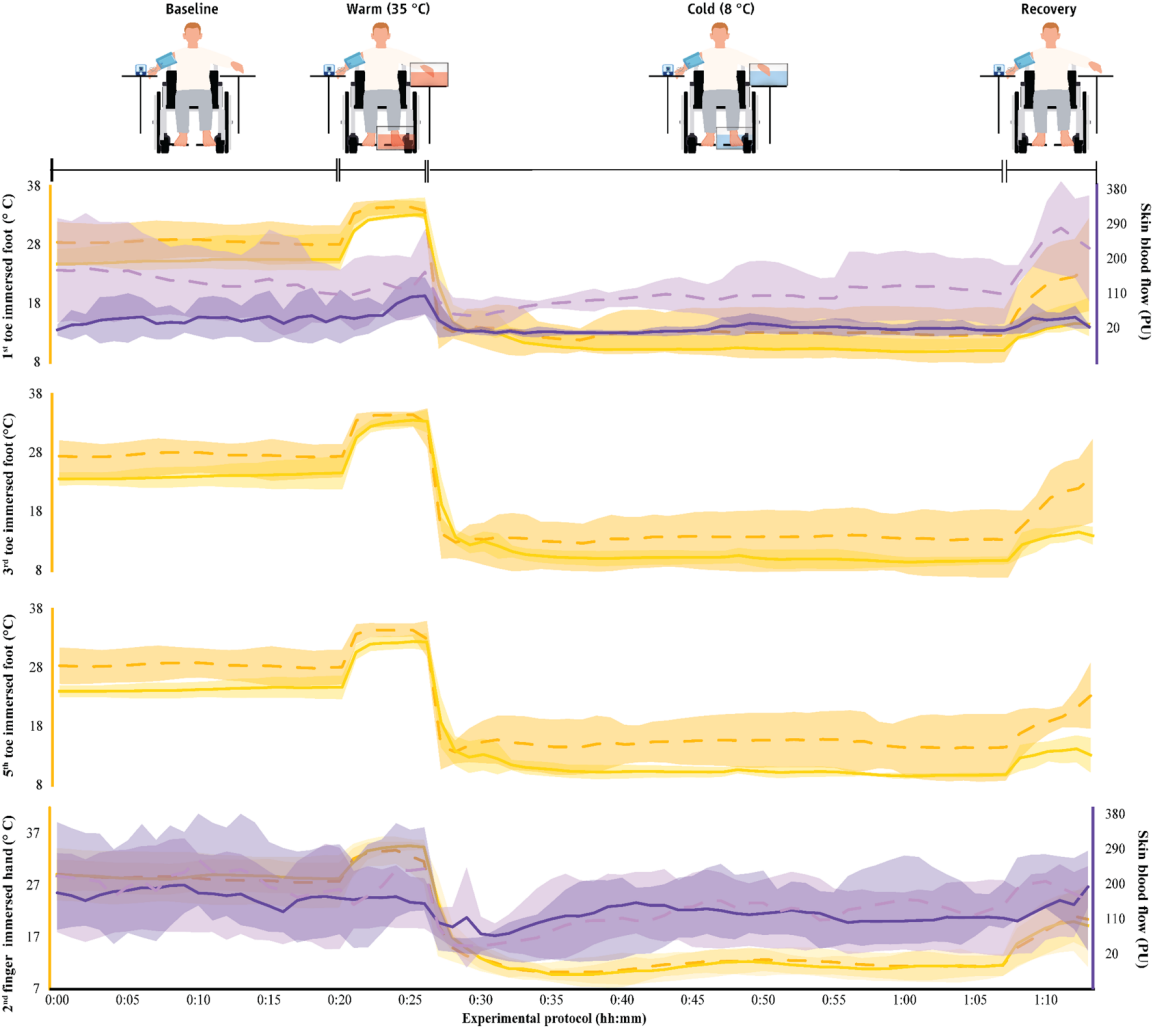
Skin temperature in the fingers and toes as well as skin blood flow (mean ± SD) in the thermoneutral environment (23 ± 1 °C) in the two groups. The first 20 min (00:00–00:20) indicate data collected during the baseline phase, the next five min (00:20–00:25) indicate responses during the warm immersion, the next 40 min (00:30–00:70) indicate responses during the cold immersion, and the final five min (00:70–00:75) show responses during the recovery phase. Finger and toe temperatures are indicated with continuous yellow lines in paraplegic participants and with dashed yellow lines in able-bodied individuals. Finger and toe skin blood flow data are indicated with continuous purple lines in paraplegic participants and with dashed purple lines in able-bodied individuals

**Fig. 6:**
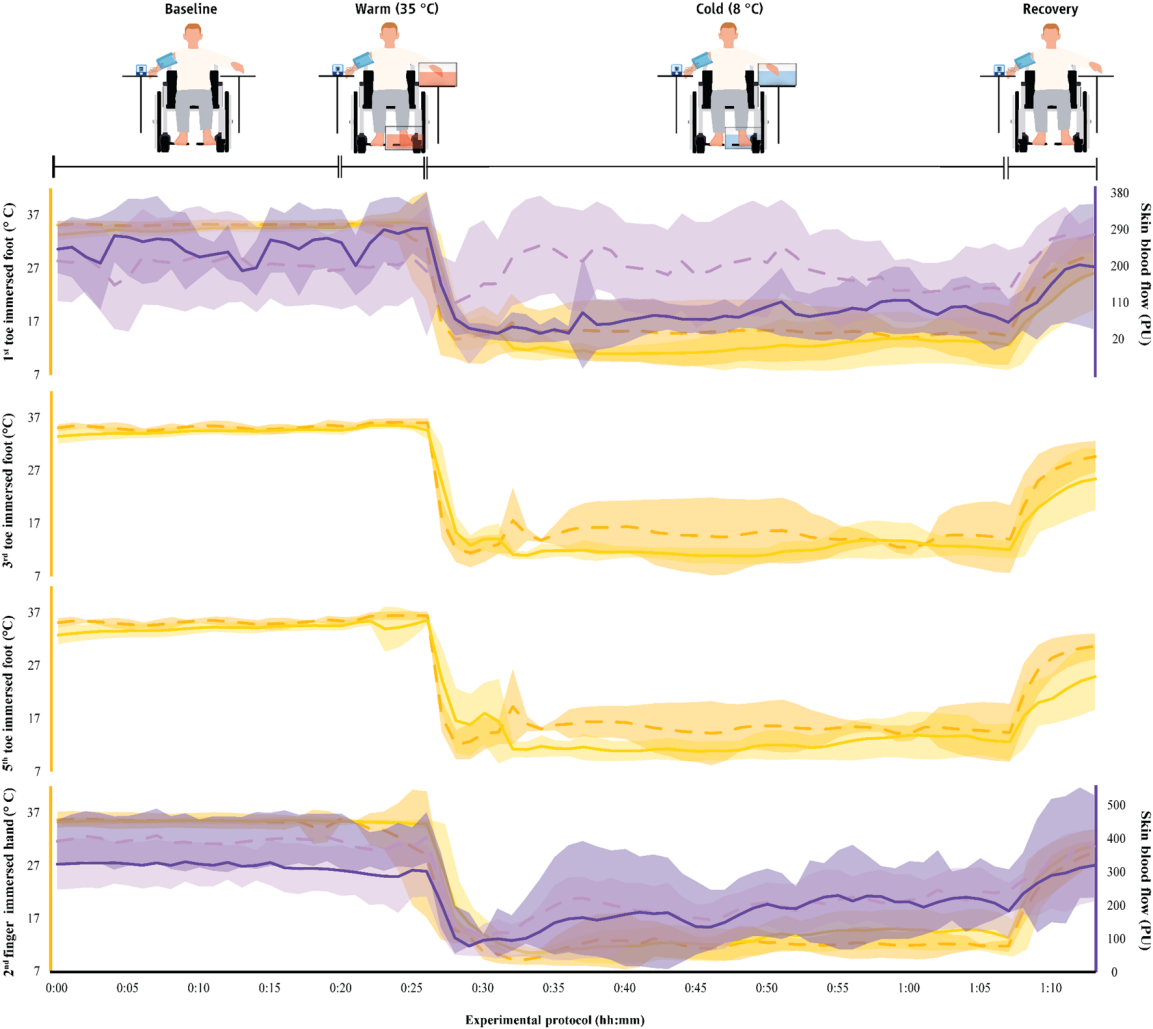
Skin temperature in the fingers and toes as well as skin blood flow (mean ± SD) in the hot environment (34 ± 1 °C) in the two groups. The first 20 min (00:00–00:20) indicate data collected during the baseline phase, the next five min (00:20–00:25) indicate responses during the warm immersion, the next 40 min (00:30–00:70) indicate responses during the cold immersion, and the final five min (00:70–00:75) show responses during the recovery phase. Finger and toe temperatures are indicated with continuous yellow lines in paraplegic participants and with dashed yellow lines in the able-bodied individuals. Finger and toe skin blood flow data are indicated with continuous purple lines in paraplegic participants and with dashed purple lines in able-bodied individuals

The characteristics of CIVD waves across groups and environments are shown in Table S1 as well as in Figs. 4, 5, and 6. For the toes, as the able-bodied participants showed no CIVD in the cool or the thermoneutral environments, we were only able to compare the CIVD characteristics between groups for the waves observed in the hot environment. We found that, in most cases, the 1st CIVD wave detected in the toes of the paraplegic group had higher *T*_min_, *T*_max_ and Δ*Τ*, as well as that it occurred later (i.e., had a later *T*_onset_) as compared to the able-bodied participants (small to very large effect sizes; Table S1). Also, the toe *T*_avg_ of the paraplegic group was higher (large to very large effect sizes). These trends between groups were also observed in the index finger (small to large effect sizes; Table S1).

### Physiological responses

**Fig. 7:**
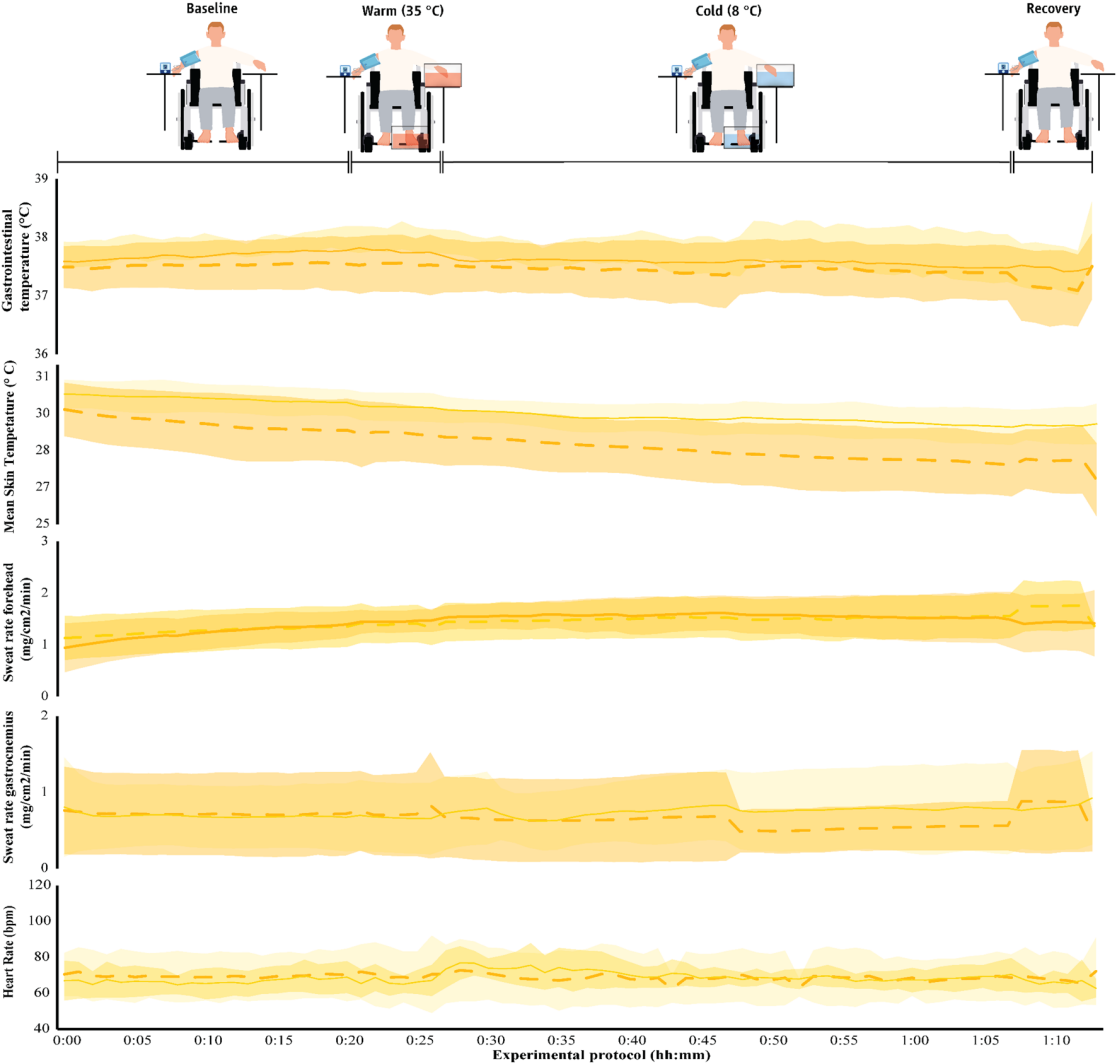
Physiological parameters (mean ± SD) during exposure to the cool environment (16 ± 1 °C) in the two groups. The first 20 min (00:00–00:20) indicate data collected during the baseline phase, the next five min (00:20–00:25) indicate responses during the warm immersion, the next 40 min (00:30–00:70) indicate responses during the cold immersion, and the final five min (00:70–00:75) show responses during the recovery phase. Continuous yellow lines indicate results for paraplegic participants and with dashed yellow lines present results for the able-bodied individuals

**Fig. 8:**
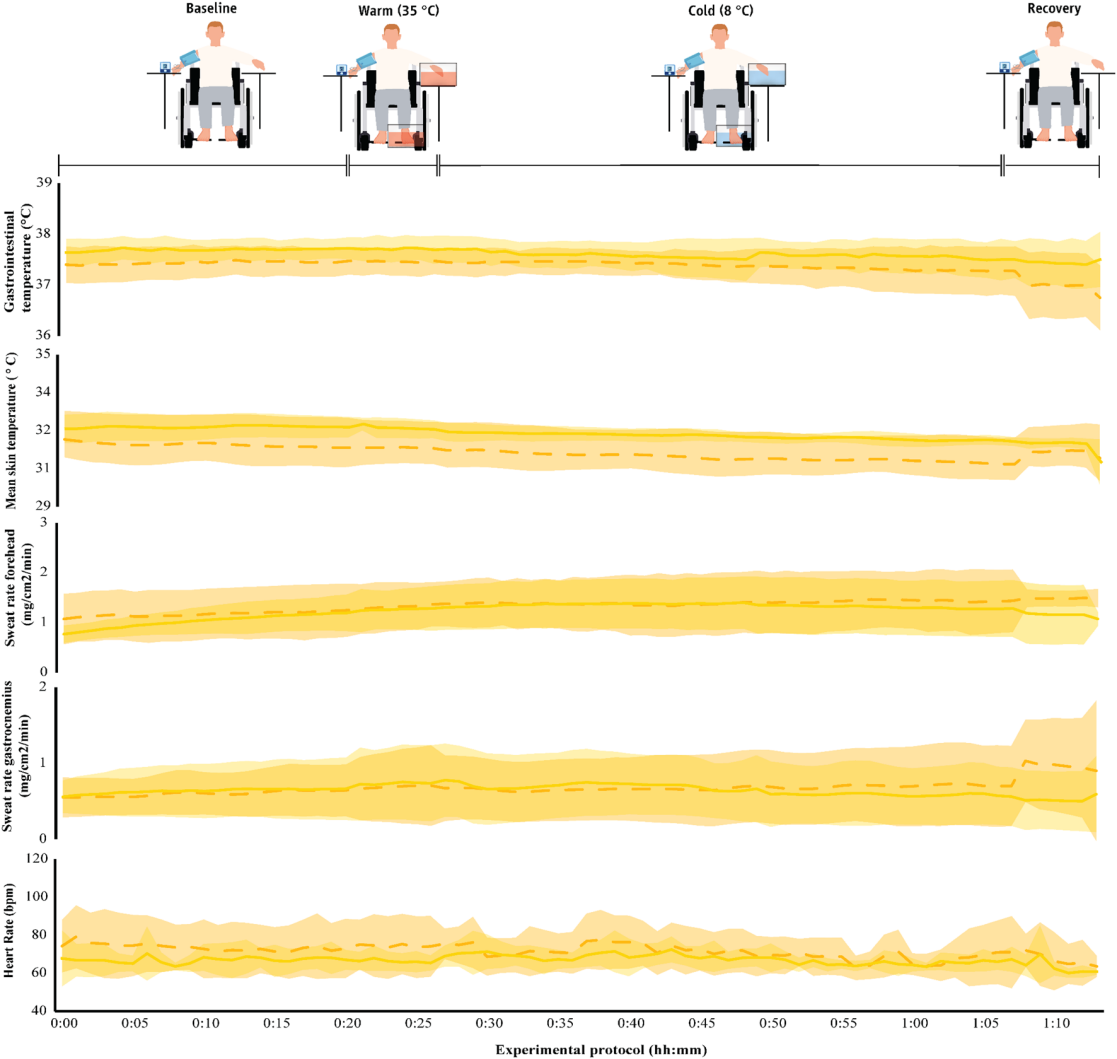
Physiological parameters (mean ± SD) during exposure to the thermoneutral environment (23 ± 1 °C) in the two groups. The first 20 min (00:00–00:20) indicate data collected during the baseline phase, the next five min (00:20–00:25) indicate responses during the warm immersion, the next 40 min (00:30–00:70) indicate responses during the cold immersion, and the final five min (00:70–00:75) show responses during the recovery phase. Continuous yellow lines indicate results for paraplegic participants and with dashed yellow lines present results for the able-bodied individuals

**Fig. 9:**
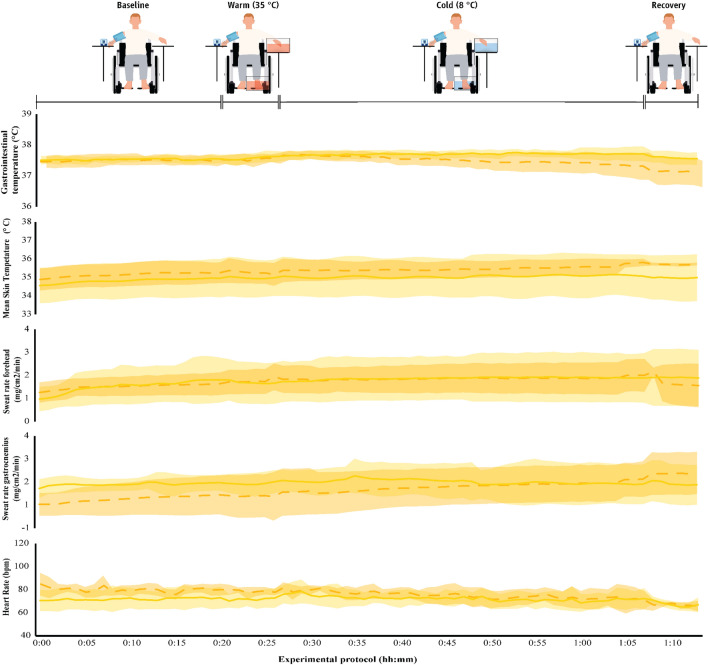
Physiological parameters (mean ± SD) during exposure to the hot environment (34 ± 1 °C) in the two groups. The first 20 min (00:00–00:20) indicate data collected during the baseline phase, the next five min (00:20–00:25) indicate responses during the warm immersion, the next 40 min (00:30–00:70) indicate responses during the cold immersion, and the final five min (00:70–00:75) show responses during the recovery phase. Continuous yellow lines indicate results for paraplegic participants and with dashed yellow lines present results for the able-bodied individuals

The physiological parameters monitored during the protocol are shown in Table S2 as well as Figs. 7, 8 and 9. In the vast majority of cases, the differences observed between groups did not reach the level of *p* < 0.008 for post hoc comparisons, yet a number of moderate or large effect sizes were detected. Specifically, the *T*_gi_ was markedly lower in the paraplegic group particularly in the cool and the thermoneutral environment (small to large effect sizes), and this effect was more pronounced during the recovery phase (Table S2). We also found that injuries at higher levels of the spinal cord (Fig. 1) were associated with lower average *T*_gi_ (*r* = 0.782, *p* = 0.038). For example, our participant with the highest level of injury (T4) demonstrated minimum *T*_gi_ at 35.80 °C in the cool environment, 35.75 °C in the thermoneutral environment, and 36.25 °C in the heat. Interestingly, this participant demonstrated repeated and marked *T*_t_ fluctuations fulfilling the criteria for CIVD reaction as it would characterize in the able-bodied population in the cool and the hot environments.

The *T*_sk_ of the participants with paraplegia was lower in the cool and the thermoneutral environments (medium to very large effect sizes), and higher in the heat (medium effect sizes). The lower *T*_sk_ of the participants with paraplegia was caused by a markedly lower thigh (medium to large effect sizes) and—even more so—leg (gastrocnemius; medium to huge effect sizes) temperature in the cool and the thermoneutral environments (Table S2). In most phases of the protocol, the temperature and SkBF in the toes of the participants with paraplegia were higher than in the able-bodied group (small to very large effect sizes; Table S2). Interestingly, we also found that injuries at higher levels of the spinal cord were associated with lower average SkBF in both the immersed (*r* = 0.891, *p* < 0.001) and the non-immersed (*r* = 0.964, *p* < 0.001) leg. The temperature of the fingers in both the immersed and the non-immersed hand tended to be relatively lower in the paraplegic group (very small to large effect sizes; Table S2). This was also true for the SkBF of the fingers in the non-immersed hand (very small to small effect sizes), as well as the SkBF of the fingers in the immersed hand in the cool and thermoneutral environments (medium effect sizes). Finally, the *T*_b_ was lower for the paraplegic group compared to the able-bodied in all three environments with large to huge effect sizes (Table S2) for all phases except one small effect size in the baseline phase in the hot environment.

The paraplegic group showed markedly lower leg sweat rate in the hot environment (small to large effect sizes; Table S2, Figs. 7, 8 and 9). No marked and/or meaningful group differences were observed in sweat rate of the forehead in any environment or of the leg (gastrocnemius) in cold and neutral environments. Heart rate was higher in the paraplegic group (very small to large effect sizes; Table S2, Figs. 7, 8 and 9). Finally, systolic blood pressure tended to be higher (very small to very large effect sizes) and diastolic blood pressure tended to be lower (very small to large effect sizes) in the paraplegic group (Table S2).

### Perceptual data

Perception of pain and distress in the hand during the cold water immersion were markedly lower for the paraplegic group (small to very large effect sizes; Table S2). Compared to the able-bodied participants, the finger tactile sensitivity of the paraplegic group tended to be higher when assessed using the Semmes–Weinstein monofilaments (small to medium effect sizes) and lower when assessed using the digital esthesiometer (very small to large effect sizes; Table S2). During the cold water immersion and the recovery, the participants with paraplegia reported being more thermally comfortable than the able-bodied individuals in the cool and the thermoneutral environments (very small to medium effect sizes), and this was reversed in the heat (medium effect sizes). Finally, in terms of thermal sensation, the paraplegic group reported feeling colder than the able-bodied group in the cool environment (small to medium effect sizes; Table S2).

## Discussion

The principal finding of the present study is the presence of a vasodilatory response in the fingers and toes of participants with paraplegia during immersion of the hands and feet in cold water; a response that is defined as a CIVD in able-bodied individuals. The elevations in the digit skin temperature undoubtedly reflect intermittently restored blood flow, but the nature of these responses—namely the frequency of their occurrence, magnitude, and duration—was different between the two groups. Should the origins of the digit skin temperature fluctuations during cold water immersion not be of the same origin in paraplegics as in able-bodied individuals, then the original premise of our study is compromised. Should the nature of the skin temperature fluctuations be similar, then the data could provide insight regarding the origin of the CIVD response, namely whether it is central or peripheral.

### Effect of paraplegia and ambient temperature on the CIVD response based on finger/toe temperature

In able-bodied participants, immersion of the hands and feet in cold water during exposure to a hot environment elicited a CIVD response in fingers and toes. The CIVD response in the toes was absent during exposure to thermoneutral and cool ambient conditions. The participants with paraplegia showed a similar reduction of the number of CIVD responses as the ambient temperature decreased from hot to thermoneutral and cool. Yet, contrary to the able-bodied participants who exhibited no CIVD responses in the toes at thermoneutral and cool ambient conditions, CIVD responses were present in the toes of participants with paraplegia.

The core body and mean skin temperatures of the participants with paraplegia was lower during the cold water immersions performed in the thermoneutral and cool environments, indicating that they had a lower body heat content during these trials compared to the able-bodied subjects. The above-mentioned observation of more frequent occurrences of toe CIVDs in the participants with paraplegia during the cool and thermoneutral conditions is contrary to what has been observed in able-bodied individuals, namely an attenuation of the CIVD response (Daanen and Ducharme 1999; Daanen et al. 1997; Flouris and Cheung 2009b; Flouris et al. 2008).

**Fig. 10:**
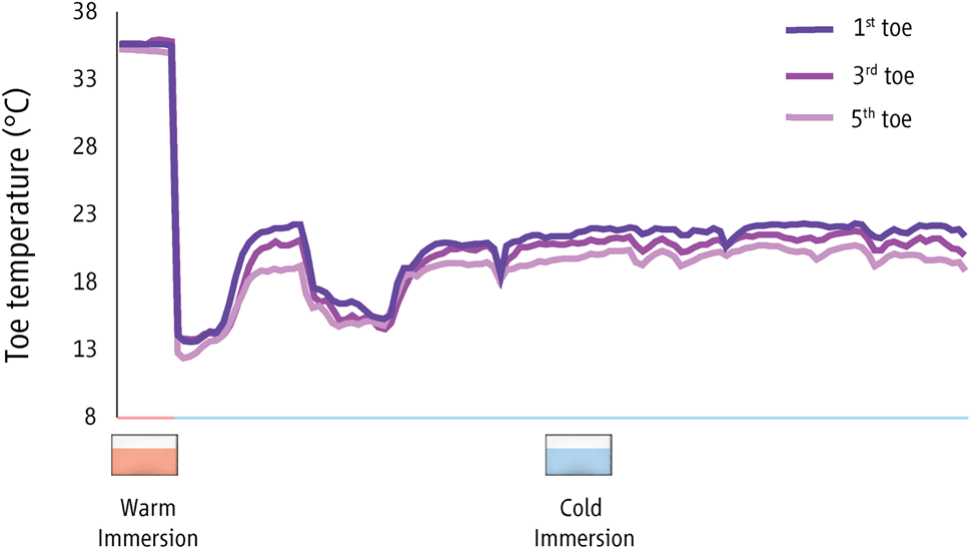
Representative data fom one participant with paraplegia (level of lesion: T4) during the warm and cold immesions while being exposed to the thermoneutral environment. In the horizontal axis, light red indicates the warm water immersion and light blue indicates the cold water immersion

In contrast to the able-bodied participants, who did not exhibit any CIVDs during exposure to thermoneutral and cool ambient conditions, the participants with paraplegia exhibited vasodilation in the toes during these trials. The lack of CIVDs in the toes of able-bodied individuals is not a novel finding (Cheung and Mekjavic 2007), and has been implicated as possibly contributing to the greater risk of freezing cold injury of the toes during exposure to extremes of cold ambient conditions. In the fingers, we found that participants with paraplegia showed a similar number of CIVDs in the thermoneutral and hot environments and fewer CIVDs in the cool environment, as compared to the able-bodied group. Since the spinal cord lesions in our paraplegic participants were lower than the level at which nerve fibers conducting afferent sensory information from the fingers and efferent information from central regions, it is not surprising that the regulation of SkBF in the upper extremity was not affected (Price and Trbovich 2018; Prévinaire et al. 2010). Therefore, the difference in the cool environment may be attributed to the lower *T*_gi_, *T*_sk_ and *T*_b_ of the participants with paraplegia, as reduced body heat content is known to significantly attenuate CIVD reactions (Daanen and Ducharme 1999; Daanen et al. 1997; Flouris and Cheung 2009b; Flouris et al. 2008). In contrast, disrupted vasomotor function of the legs is due to the abolished or severely limited sympathetic outflow below the level of the spinal cord lesion (Price and Trbovich 2018; Nicotra et al. 2004; Nash et al. 1996). We had hypothesized that participants with paraplegia would demonstrate limited or no CIVD responses in their toes, but our findings show large SkBF fluctuations in the toes of these individuals and that these reactions that would have been described as CIVD in able-bodied participants were more prevalent in the paraplegic group than in able-bodied participants. These results are in line with a number of previous studies reporting marked increases and/or fluctuations in SkBF in the legs of people with paraplegia as a response to thermal stimuli (Scheel-Sailer et al. 2020; Taylor et al. 1993; Price and Trbovich 2018; Cooper et al. 1957; Tsai et al. 1980). In fact, a previous study showed larger SkBF fluctuations of the toes in paraplegic participants compared to able-bodied individuals in a thermoneutral environment (Deitrick et al. 2007), mirroring our findings. Moreover, careful examination of the individual data revealed that these fluctuations in the toe temperature of participants with paraplegia were almost symmetrical in amplitude and synchronous in all toes (Fig. 10). This is contrary to previous studies, which showed that CIVDs are typically asymmetrical in amplitude and asynchronous in across both fingers (Cheung and Mekjavic 2007) and toes (Reynolds et al. 2007). Another important observation is that the participants with paraplegia that did not reveal fluctuations in toe skin temperature / blood flow, demonstrated none or very few CIVDs in the fingers (0–3 finger CIVDs in total) compared to the participants with paraplegia who showed fluctuations in toe skin temperature / blood flow (3–5 finger CIVDs in total). Interestingly, these CIVDs in the fingers of the latter three participants were almost identical in amplitude and simultaneous for all fingers, as was the case in their toes.

### Effect of paraplegia and ambient temperature on skin blood flow and CIVD

The most pertinent issues with regards to the observed indirect evidence (i.e., skin temperature) of the skin blood flow responses in the fingers and toes of participants with paraplegia are: can the responses be defined as CIVDs in the same manner as in the able-bodied population, or are these responses of a different nature and origin, specific to individuals with spinal cord injury at a level that affects the innervation of the toes and not the fingers?

Since SkBF fluctuations were observed in some individuals in our paraplegic group and not in others, it is important to consider the potential impact of the level of injury, since the control of SkBF of the lower extremity is mediated from T10 to L2 (Prévinaire et al. 2010), while sensory innervation of the skin in the lower extremities is mediated from L1 to S4 (Kirshblum et al. 2011). The three paraplegic participants in our study who demonstrated marked SkBF, and *T*_t_ fluctuations had lesions localized at thoracic level (T4, T11, and T12), while the participants who did not demonstrate any such fluctuations had lesions at levels T9, T11, T12, and L1. This is unexpected, as the latter individuals who had lower (T9, L1) or the same level of injury (T12, T11) would be more likely to show thermal-induced fluctuations in SkBF and *T*_t_ (Cooper et al. 1957; Price and Trbovich 2018). Also, the paraplegic participants who demonstrated marked vasodilations had lower *T*_gi_ in the cool (36.3 ± 0.2 °C vs. 37.0 ± 0.4 °C) and the hot (36.9 ± 0.3 °C vs. 37.1 ± 0.2 °C) environments and relatively similar *T*_gi_ (37.0 ± 0.1 °C vs. 36.8 ± 0.4 °C) in the thermoneutral environment compared to our paraplegic participants who did not demonstrate any CIVD. Again, this is counterintuitive and contradicts previous CIVD findings in able-bodied individuals, namely that attenuated body heat content reduces the occurrence of CIVD (Daanen and Ducharme 1999; Daanen et al. 1997; Flouris and Cheung 2009b; Flouris et al. 2008). Also, these fluctuations were more frequent in the thermoneutral and cool environments instead of the hot environment. The able-bodied participants showed a complete lack of such responses in the thermoneutral and cool environments, similarly to a previous study (Cheung and Mekjavic 2007), and much higher occurrence in the hot environment. Taken together, these results suggest that, while the T_t_ responses of the paraplegic group technically fulfilled the criteria for CIVD, it is unlikely that they reflect the same phenomenon as that originally reported by Lewis in 1930 (Lewis 1930). A potential explanation for the fluctuations in SkBF, and thus *T*_t_, of participants with paraplegia in our study may be that they are caused by mechanical stimuli such as bladder stretching which can cause vasomotor impulses to pass down the sympathetic vasomotor fibers to the foot (Cooper et al. 1957). The counterintuitive appearance of these SkBF and *T*_t_ fluctuations in the cool environment confirms previous evidence suggesting that the origin of the vasomotor impulses in paraplegia is not modified by changing the temperature of the spinal cord and is, therefore, not related to thermoregulation (Cooper et al. 1957). Yet, it is important to state that spinal cord temperature was not measured in our study.

### The possible implication of muscular and vascular atrophy

Compared to able-bodied individuals, participants with paraplegia demonstrated lower *T*_sk_ in the cool and the thermoneutral environments, and higher *T*_sk_ in the heat, in line with previous studies (Price and Campbell 1997; Price and Trbovich 2018). As expected, these differences in *T*_sk_ stemmed primarily from the lower thigh and—even more so—lower leg skin temperatures. Previous studies have attributed these effects to reduced muscle activity of paraplegic individuals, which leads to attenuated metabolic heat production below the level of the lesion, as well as to an atrophied vascular system below the level of the spinal cord lesion, resulting in a reduced convective heat transfer from the muscle to the skin (Hopman et al. 1993; Nash et al. 1996). Importantly, the marked differences observed in the skin temperature of the thigh and lower leg between able-bodied individuals and participants with paraplegia were blunted when the data are presented as *T*_sk_. This finding coincides with previous work (Price and Campbell 1997; Price and Trbovich 2018), highlighting that commonly used equations to calculate *T*_sk_ may mask regional differences in skin temperature and are inappropriate for people with paraplegia. Since the mean skin temperature is not representative for people with paraplegia, *T*_b_, which takes into account both core body and mean skin temperature may be a good method for measuring body heat context in paraplegic participants because it might give a better understanding of the whole-body heat content. Mean body temperature is a very good indicator of the body's heat content in the able-bodied population, and it is known from the literature that body heat content significantly influences CIVD responses (Flouris et al. 2008). Our paraplegic participants had lower *T*_b_, during all environment and phases.

### The contribution of central and peripheral mechanisms in the etiology of CIVD

The present study was designed with the view of contributing to the resolution of the issue regarding the contribution of central and peripheral (local) mechanisms in the initiation of CIVDs during immersion of either the hands or feet in cold water. We considered that our approach of comparing the CIVD response in fingers and toes of able-bodied and paraplegic individuals would provide evidence in support of one or the other. The evidence generated by this study has, perhaps, generated more questions than it has resolved. Indeed, the absence of CIVDs in toes of able-bodied individuals in thermoneutral and cool ambient conditions, but not in the toes of the paraplegics, would favor a local mechanism. As discussed above, the observed skin temperature fluctuations in individuals with paraplegia may have been initiated by nonthermal factors (e.g., bladder stretching), thus confounding our ability to discern the origin of the responses. In this sense, our results favor a central origin of the CIVD response (Flouris and Cheung 2009a, b, 2010; Flouris et al. 2008; Mekjavic et al. 2008), which was recently supported by a study showing that whole-body heat acclimation can augment the CIVD response, whereas daily immersion of the hand in warm water does not (Ciuha et al. 2021). Conducting a similar study with paraplegic participants, namely comparing the CIVD responses after heat acclimation with those observed after daily immersions of the hands and feet in warm water, may help to further confirm the contribution of central and peripheral factors in the regulation of digit skin blood flow in paraplegic participants.

### Limitations

We did not monitor bladder stretching or urine production, which could have provided insightful information with regards to the unexplained fluctuations in skin temperature and blood flow of individuals with paraplegia. Also, it is important to note that we did not directly assess sympathetic vasomotor control in the fingers and toes of our participants. Instead, we assumed a lack of sympathetic outflow to the toes of our paraplegic participants and a normal such function in our able-bodied individuals. Future studies should address this gap and should aim to assess CIVD in a larger sample of people with paraplegia. In this regard, it is crucial to ensure that the recruited participants have experienced the spinal cord lesion for > 6 months, as this is known to affect vasomotor and sudomotor function below the level of the lesion. In our study, the time since injury ranged from 2 to 40 years. It would be interesting to explore potential links between the occurrence and characteristics of CIVD and the time since injury, yet our sample size did not allow such analyses. However, we should note that the participants in our paraplegic group that presented these fluctuations were the ones that were injured most recently (i.e., approximately the past two years).

It could be argued that the analysis of the area under the curve of the CIVD responses would provide a more integrative result, of more practical relevance than that of a comparison of the individual characteristics of the CIVD response (Wickham et al. 2021). However, the vast majority of studies investigating CIVD have done so by examining, and reporting, the individual CIVD characteristics. Thus, for the purpose of validating the observed responses observed in able-bodied individuals and comparing the CIVD responses observed in participants with paraplegia, it was deemed more relevant to conduct the comparison of the responses based on the classical approach in analyzing the CIVD responses. Finally, it is worth noting that the lack of heterogeneity and group differences in CIVD reactions in the hands is likely due to the single measurement site, contrary to the toes where we used three measurement sites and found significant group differences. As this is the first study that investigated the phenomenon of CIVD in people with paraplegia, we focused our attention on the reactions of the toes.

### Practical implications

In regions with colder climates, it is often rare for individuals with paraplegia to venture outdoors during periods of extreme cold. Without feedback regarding the thermal status of their feet, they cannot appropriately behaviourally thermoregulate (i.e., adding more insulation, removing themselves from the cold, etc.). The extent to which CIVD responses contribute to the prevention of non-freezing and freezing cold injury in able-bodied individuals remains unresolved (Daanen and van der Struijs 2005; Keramidas et al. 2010) although CIVDs of low magnitude and short duration are unlikely to have a significant effect. These characteristics are also observed in the skin temperature changes of individuals with paraplegia. Thus, it is unlikely that the skin temperature fluctuations observed in people with paraplegia could provide any benefit against cold injury. Cold injury in the lower extremities of individuals with paraplegia can have serious consequences. For instance, there are anecdotal cases from North America, where a day outing on a snowmobile resulted in cold injuries in people with paraplegia, ultimately requiring amputation of the frostbitten limbs. Furthermore, the delay in time to initiation of these fluctuations to start (i.e., long onset time) in the paraplegic group may also be a proof that these waves do not have a protective role against cold injuries.

## Conclusions

In conclusion, our findings demonstrated considerable inter-individual variability in CIVD responses in both the paraplegic and able-bodied groups. We observed vasodilatory responses in the fingers and toes of participants with paraplegia during immersion of the hands and feet in cold water; a response that is defined as a CIVD in able-bodied individuals. While these responses in the toes of the paraplegic participants technically fulfilled the criteria for CIVD, their characteristics are counterintuitive, and it is unlikely that they reflect the phenomenon of CIVD observed in able-bodied individuals. Taken together, the evidence presented herein favor the contribution of central over peripheral factors in relation to the origin and/or control of CIVD. Finally, there are no standardized criteria to identify CIVD for people with paraplegia or able-bodied individuals. The only well-accepted criterion is the continuous increase of at least 1 °C in finger/toe skin temperature. This knowledge gap should be addressed by future studies, potentially adopting the Delphi method, to provide more clarity to the CIVD phenomenon.

## Supplementary Information

Below is the link to the electronic supplementary material.

Supplementary file1 (PDF 303 kb)

## Data Availability

The data that support the findings of this study are available from the corresponding author, upon reasonable request.

## Abbreviations

CIVD: Cold-induced vasodilation
*T*_gi_: Gastro-intestinal temperature
*T*_sk_: Mean skin temperature
*T*_b_: Mean body temperature
*T*_f_: Finger temperature
*T*_t_: Toe temperature
SkBF: Skin blood flow
*N*: Number of waves
*T*_min_: Minimum temperature
*T*_max_: Maximum temperature
*T*_onset_: Onset time
*T*_peak_: Peak time
*T*_avg_: Average temperature
Δ*T*: Temperature amplitude

## Declarations

### Disclaimer

The opinions or assertions contained herein are the private views of the authors and are not to be construed as official or as reflecting the views of the U.S. Army, the Department of Defense, or the U.S. Government. Approved for public release; distribution is unlimited. Citations of commercial organizations and trade names in this report do not constitute an official U.S. Department of the Army endorsement or approval of the products or services of these organizations.
