# Supplementary material for: Central versus peripheral mechanisms of cold-induced vasodilation: a study in the fingers and toes of people with paraplegia
Source: Eur J Appl Physiol. 2023 Apr 2;123(8):1709–26. doi: 10.1007/s00421-023-05175-7 (PMC10363085; doi:10.1007/s00421-023-05175-7)
Supplement: Supplementary file 1 — Supplementary file1 (PDF 303 kb) [file 421_2023_5175_MOESM1_ESM.pdf]

Central versus peripheral mechanisms of cold induced vasodilation: a study in the fingers and toes of people with paraplegia

Lydia Tsoutsoubi[1], Leonidas G. Ioannou[1], Billie K. Alba[2], Stephen S. Cheung[3], Hein A. Daanen[4], Igor B. Mekjavic[5,6], Andreas D. Flouris[1]*

[1]FAME Laboratory, Department of Physical Education and Sport Science, University of Thessaly, Trikala, Greece.

[2]Thermal and Mountain Medicine Division, U.S. Army Research Institute of Environmental Medicine, Natick, MA 01760, USA

[3]Department of Kinesiology, Brock University, St. Catharines, Ontario, Canada

[4]Faculty of Behavioural and Movement Sciences, Vrije Universiteit Amsterdam, Amsterdam, The Netherlands

[5]Department of Automation, Biocybernetics and Robotics, Józef Stefan Institute, SI-1000 Ljubljana, Slovenia

[6]Department of Biomedical Physiology and Kinesiology, Simon Fraser University, Burnaby, British Columbia, V5A 1S6 Canada

Corresponding author:

Andreas D. Flouris

email: andreasflouris@gmail.com

FAME Laboratory, Department of Physical Education and Sport Science

University of Thessaly

Karies, Trikala, 42100, Greece

Tel: +30 2431 047 072

Table S1. Characteristics of CIVD waves in paraplegic (PAR) and able-bodied (AB) individuals in the tested environments across fingers and toes.

			N	Minimum (°C)	Maximum (°C)	Onset time (mm: ss)	Peak time (mm: ss)	Average (°C)	Amplitude (°C)
1st toe immersed foot	**Wave 1**								
	Cold	PAR	1	12.0	22.7	03:01	06:43	21.9	10.7
		AB	0						
	Neutral	PAR	1	9.3	12.6	09:26	05:40	11.2	3.3
		AB	0						
	Hot	PAR	3	12.8±2.5	17.5±5.3	21:09±16:21	05:49±02:01	16.4±5.9	4.7±2.8
		AB	4	10.0±1.5	13.1±3.3	14:38±08:41	08:13±01:04	10.7±1.2	3.1±1.9
	**Wave 2**								
	Cold	PAR	0						
		AB	0						
	Neutral	PAR	0						
		AB	0						
	Hot	PAR	1	11.2±0.1	14.4±0.8	23:44±04:40	04:50±00:14	13.0±0.1	3.2±0.6
		AB	1	8.5	11.0	25:07	12:00	10.3	2.5
3rd toe immersed foot	**Wave 1**								
	Cold	PAR	1	10.8	17.5	06:24	03:00	16.0	6.7
		AB	0						
	Neutral	PAR	2	9.6±1.3	12.9±0.3	06:53±03:36	05:23±01:50	10.6±0.0	3.3±1
		AB	0						
	Hot	PAR	3	9.9±0.9	16.6±4.6	20:42±16:22	07:15±02:00	14.7±4.1	6.6±4.3
		AB	3	10.6±0.4	13.1±1.4	11:05±04:02	04:20±02:11	11.6±1.1	2.5±1.7
	**Wave 2**								
	Cold	PAR	0						
		AB	0						
	Neutral	PAR	0						
		AB	0						
	Hot	PAR	1	8.3	13.5	19:46	10:40	12.0	5.2
		AB	2	8.9±0.7	15.7±6.1	22:55±02:09	07:30±03:04	12.0±1.2	6.8±6.8
	**Wave 3**								
	Cold	PAR	0						
		AB	0						
	Neutral	PAR	0						
		AB	0						
	Hot	PAR	0						
		AB	1	9.7	10.8	35:07	02:00	11.2	1.0
5th toe immersed foot	**Wave 1**								
	Cold	PAR	1	11.1	18.2	06:24	01:40	16.0	7.1
		AB	0						
	Neutral	PAR	1	9.4	15.0	08:46	08:40	12.3	5.7
		AB	0						
	Hot	PAR	3	10.7±1.8	17.3±2.6	19:49±15:52	07:42±02:11	15.2±3.0	6.6±1.4
		AB	4	10.1±1.6	13.2±2.7	16:14±07:41	05:30±03:07	11.4±1.8	3.2±2.4
	**Wave 2**								
	Cold	PAR	0						
		AB	0						
	Neutral	PAR	0						
		AB	0						
	Hot	PAR	1	8.8	15.0	19:46	04:00	13	6.3
		AB	2	12.1±0.1	16.2±4.5	30:23±06:35	05:10±04:00	12.7±1.8	4.2±4.3

Table S1. Characteristics of CIVD waves in paraplegic (PAR) and able-bodied (AB) individuals in the tested environments across fingers and toes.

			N	Minimum (°C)	Maximum (°C)	Onset time (mm: ss)	Peak time (mm: ss)	Average (°C)	Amplitude (°C)
2nd finger immersed hand	**Wave 1**								
	Cold	PAR	2	9.4±2.3	12.9±3.8	10:53±01:14	06:50±00:14	11.3±2.4	3.6±1.5
		AB	6	8.1±0.8	10.7±2.1	11:20±05:23	07:46±02:55	9.0±1.1	2.6±1.4
	Neutral	PAR	4	8.6±2.2	14.2±2.9	09:38±02:24	11:00±03:40	11.1±1.9	5.6±2.5
		AB	4	9.4±1.2	11.1±2.7	08:55±02:07	08:08±01:22	9.3±2.2	3.6±1.6
	Hot	PAR	5	10.1±2.1	15.6±3.9	11:47±08:26	08:56±07:18	12.3±1.9	5.5±4.2
		AB	4	9.7±0.9	15.3±2.9	09:29±03:44	07:55±05:10	12.8±1.8	5.6±2.7
	**Wave 2**								
	Cold	PAR	1	9.4	11.5	20:46	07:20	9.6	2.1
		AB	1	10.2	12.9	30:21	03:20	11.7	2.7
	Neutral	PAR	1	12.2	14.3	22:00	04:00	12.1	2.1
		AB	4	10.3±2.1	13.5±3.1	28:44±05:12	06:20±03:57	11.6±2.2	3.2±1.7
	Hot	PAR	3	10.5±1.6	15.6±2.6	19:04±01:29	06:53±02:50	12.5±1.4	5.1±2.7
		AB	3	10.4±1.0	13.4±1.2	23:52±02:41	05:13±01:04	12.1±1.4	2.9±0.2
	**Wave 3**								
	Cold	PAR	0						
		AB	0						
	Neutral	PAR	0						
		AB	0						
	Hot	PAR	1	9.6	15.2	27:42	07:20	13.2	5.7
		AB	0						

Notes:

Minimum corresponds to the minimum value prior the CIVD.
Maximum corresponds to the maximum value during the CIVD.
Onset time correspond to the time of the minimum value minor the start time of the cold immersion.
Peak time correspond to the time of the minimum value minor the time of the maximum value.
Average corresponds to the mean values of the last 35 minutes of the cold immersion.
Amplitude corresponds to the difference of the maximum minor the minimum temperature.

Table S2. Physiological and perceptual parameters in paraplegic (PAR) and able-bodied (AB) individuals in the tested environments. Effect sizes indicate between group comparisons.

			Baseline	Warm immersion	Cold immersion	Recovery	Effect size (d)			
							Base	WI	CI	Rec
Gastro-intestinal temperature (°C)	Cold	PAR	37.02±0.41	37.05±0.47	36.90±0.48	36.63±0.63	0.54	0.61	0.45	1.04
		AB	37.19±0.17	37.27±0.21	37.07±0.27	37.13±0.26				
	Neutral	PAR	36.94±0.31	36.96±0.27	36.88±0.32	36.50±0.64	0.94	0.98	0.63	1.16
		AB	37.19±0.22	37.22±0.24	37.07±0.27	37.10±0.37				
	Hot	PAR	37.01±0.17	37.03±0.16	37.01±0.25	36.64±0.44	0.16	0.27	0.99	1.69
		AB	37.03±0.10	37.08±0.15	37.20±0.12	37.18±0.12				
Mean skin temperature (°C)	Cold	PAR	29.13±1.22	28.71±1.19	27.97±1.16	27.60±1.31	1.04	1.14	1.43	1.23
		AB	30.12±0.58	29.78±0.57	29.28±0.57	28.94±0.78				
	Neutral	PAR	31.45±0.96	31.32±0.85	30.94±0.82	31.19±0.82	0.92	1.17	1.35	0.62
		AB	32.15±0.49	32.10±0.38	31.76±0.22	31.56±0.20				
	Hot	PAR	35.12±0.67	35.30±0.61	35.46±0.48	35.71±0.07	0.52	0.55	0.70	0.79
		AB	34.67±1.02	34.82±1.07	34.87±1.09	34.88±1.49				
Mean body temperature (°C)	Cold	PAR	34.18±0.54	34.05±0.56	33.69±0.50	33.38±0.61	1.15	1.25	1.47	1.57
		AB	34.64±0.19	34.57±0.22	34.27±0.25	34.18±0.38				
	Neutral	PAR	34.96±0.44	34.93±0.39	34.74±0.37	34.59±0.70	1.23	1.44	1.35	1.00
		AB	35.38±0.20	35.38±0.20	35.16±0.23	35.11±0.25				
	Hot	PAR	35.87±1.16	34.16±0.52	33.80±0.45	33.58±0.57	0.42	1.70	2.30	1.58
		AB	36.23±0.41	34.93±0.39	34.74±0.37	34.59±0.70				
Arm temperature (°C)	Cold	PAR	31.66±0.72	31.14±0.80	30.17±0.99	29.16±1.18	0.83	0.50	0.17	0.83
		AB	31.09±0.64	30.78±0.66	30.32±0.73	29.93±0.56				
	Neutral	PAR	32.98±0.75	32.82±0.62	32.54±0.73	32.48±1.10	0.05	0.65	0.55	0.31
		AB	33.02±0.48	33.13±0.28	32.85±0.30	32.74±0.36				
	Hot	PAR	36.07±0.50	36.06±0.63	36.19±0.43	36.25±0.29	0.87	0.35	0.42	0.09
		AB	35.64±0.48	35.87±0.44	35.99±0.50	36.28±0.30				
Chest temperature (°C)	Cold	PAR	29.97±2.20	29.76±2.19	29.25±2.47	29.00±3.13	0.32	0.37	0.44	0.54
		AB	30.52±1.04	30.41±1.19	30.12±1.35	30.40±1.86				
	Neutral	PAR	32.63±1.22	32.52±1.19	32.14±1.18	31.96±1.16	0.15	0.02	0.19	0.36
		AB	32.48±0.73	32.49±0.63	32.31±0.45	32.27±0.35				
	Hot	PAR	35.83±0.46	35.87±0.41	35.91±0.33	35.99±0.32	0.89	0.84	0.85	0.78
		AB	34.48±2.09	34.55±2.17	34.51±2.30	34.00±3.60				
Leg temperature (°C)	Cold	PAR	25.11±3.35	24.61±3.42	24.03±2.54	24.27±1.36	1.54	1.47	1.80	1.79
		AB	28.85±0.75	28.25±0.73	27.51±1.00$^{0.006}$	26.29±0.84				
	Neutral	PAR	28.30±1.59	28.26±1.55	27.67±1.81	28.47±1.05	2.24	2.10	1.82	1.42
		AB	31.10±0.77$^{0.006}$	30.83±0.77	30.14±0.65	29.57±0.34				
	Hot	PAR	33.30±1.72	33.88±1.34	34.27±1.17	34.76±0.25	0.74	0.48	0.17	0.83
		AB	34.21±0.34	34.37±0.49	34.43±0.53	34.54±0.28				
Thigh temperature (°C)	Cold	PAR	28.06±1.77	27.57±1.69	26.69±1.45	26.52±1.29	0.86	0.91	1.20	0.93
		AB	29.33±1.11	28.86±1.11	28.24±1.10	27.92±1.69				
	Neutral	PAR	30.53±1.91	30.36±1.77	30.03±1.46	30.79±1.27	0.63	0.66	0.81	0.07
		AB	31.41±0.47	31.22±0.44	30.94±0.62	30.71±0.90				
	Hot	PAR	33.60±1.79	33.87±1.70	34.23±1.49	35.41±0.21	0.59	0.57	0.37	0.70
		AB	34.50±1.21	34.71±1.22	34.76±1.37	34.45±1.93				
1st toe immersed foot (°C)	Cold	PAR	23.61±4.85	32.82±2.65	13.11±3.61	15.90±5.85	0.69	0.40	1.24	1.09
		AB	21.15±1.41	31.79±2.48	9.88±0.74	11.36±0.48				
	Neutral	PAR	28.53±3.19	34.19±1.16	13.07±3.65	20.76±5.92	1.12	1.25	0.79	1.85
		AB	25.32±2.51	32.44±1.60	10.98±0.82	12.80±1.37				
	Hot	PAR	35.15±0.75	34.13±3.95	14.98±4.69	25.97±4.83	2.04	0.43	0.57	1.45
		AB	33.98±0.31	35.36±0.82	12.72±3.03	19.95±3.36				
3rd toe immersed foot (°C)	Cold	PAR	20.57±3.51	32.80±2.77	12.44±1.82	16.36±3.47	0.37	0.23	1.78	1.81
		AB	19.57±1.55	32.20±2.42	9.98±0.70	11.79±0.84				
	Neutral	PAR	27.63±2.98	34.26±1.23	13.54±4.52	19.78±5.12	1.47	1.27	0.89	1.65
		AB	24.02±1.80	32.76±1.14	10.64±1.04	13.47±1.77				
	Hot	PAR	29.84±10.58	35.95±0.52	14.63±3.74	26.62±4.06	0.57	1.24	0.83	1.27
		AB	34.13±0.81	35.37±0.41	12.31±1.25	21.17±4.51				
5th toe immersed foot (°C)	Cold	PAR	20.97±3.76	32.67±2.81	12.37±2.56	15.36±2.57	0.27	0.45	0.63	1.48
		AB	20.17±1.77	31.61±1.68	10.97±1.88	12.02±1.88				
	Neutral	PAR	28.41±2.87	34.26±1.14	15.05±4.17	19.33±3.23	1.81	1.98	1.46	2.26
		AB	24.34±1.37	31.98±1.16	10.70±0.67	13.07±2.23				
	Hot	PAR	29.85±10.61	36.25±0.54	15.20±2.80	27.82±3.50	0.50	1.95	1.31	1.48
		AB	33.65±1.08$^{0.002}$	35.14±0.59	11.97±2.09	21.22±5.27				

Table S2. Physiological and perceptual parameters in paraplegic (PAR) and able-bodied (AB) individuals in the tested environments. Effect sizes indicate between group comparisons.

Parameter	Environment	Group	Baseline	Warm immersion	Cold immersion	Recovery	Effect size (d) Base	WI	CI	Rec
1st toe non immersed foot (°C)	Cold	PAR	22.52±3.72	21.51±3.81	18.93±4.51	19.68±5.15	0.25	0.19	0.24	0.75
		AB	21.80±1.46	20.98±1.07	18.15±1.03	16.88±1.12				
	Neutral	PAR	28.70±2.63	28.20±2.48	26.57±2.95	26.84±3.82	1.21	0.80	0.97	1.65
		AB	25.46±2.72	25.89±3.22	23.82±2.74	21.99±1.64				
	Hot	PAR	34.91±0.90	34.74±0.96	34.45±0.59	34.54±0.39	0.92	0.18	0.74	0.83
		AB	34.15±0.74	34.60±0.51	33.86±0.94	33.99±0.84				
2nd finger immersed hand (°C)	Cold	PAR	18.94±3.15	29.59±2.11	10.96±1.44	13.23±2.50	0.44	0.50	0.17	0.18
		AB	20.60±4.39	30.72±2.39	11.16±1.02	13.60±1.35				
	Neutral	PAR	28.31±4.85	32.98±2.61	11.97±1.90	18.53±4.28	0.08	0.42	0.13	0.78
		AB	28.65±2.94	33.83±1.19	11.73±1.68	16.03±1.43				
	Hot	PAR	35.49±1.37	32.67±5.76	12.16±1.33	25.15±2.52	0.11	0.64	0.33	0.35
		AB	35.37±0.56	35.30±0.34	12.80±2.41	26.46±4.59				
2nd finger non immersed hand (°C)	Cold	PAR	18.69±2.05	17.35±0.92	16.33±0.51	16.37±0.36	0.64	0.75	0.83	0.45
		AB	20.78±4.16	19.58±4.12	17.25±1.48	16.22±0.29				
	Neutral	PAR	27.72±3.82	26.90±3.73	24.97±2.82	24.56±2.95	0.21	0.16	0.14	0.86
		AB	28.43±2.98	27.41±2.66	24.62±2.20	22.75±0.48				
	Hot	PAR	35.78±0.39	35.58±0.62	35.21±0.46	34.96±0.58	0.87	0.32	0.35	0.29
		AB	35.36±0.56	35.40±0.49	35.04±0.55	35.12±0.51				
SkBF 1st toe immersed foot (PU)	Cold	PAR	84.52±135.2	96.41±118.80	101.43±76.66	156.01±112.07	0.79	0.63	1.52	1.39
		AB	8.88±4.91	41.46±29.56	18.69±8.41	44.98±10.93				
	Neutral	PAR	145.04±101.06	107.74±87.49	124.28±81.43	234.98±68.39	1.26	0.47	1.85	4.07
		AB	43.70±52.86	75.44±40.12	17.09±9.06	36.11±10.38				
	Hot	PAR	186.03±91.36	189.72±91.42	176.62±102.86	250.28±49.57	0.75	0.60	1.27	1.08
		AB	244.52±61.98	249.98±109.07	79.42±33.58	145.69±127.46				
SkBF 1st toe non immersed foot (PU)	Cold	PAR	83.11±102.23	57.49±59.38	39.86±36.02	128.51±107.39	0.98	1.00	0.97	0.57
		AB	12.11±4.82	15.11±9.92	14.32±9.29	79.53±54.92				
	Neutral	PAR	63.23±49.12	58.82±56.57	133.56±210.50	138.54±85.21	1.12	0.87	0.77	0.34
		AB	23.97±7.72	22.67±15.50	18.15±10.15	162.83±54.85				
	Hot	PAR	140.46±110.67	157.02±114.36	151.08±91.79	333.49±155.23	0.49	0.35	0.05	0.35
		AB	188.56±82.74	192.65±86.05	155.48±76.68	286.31±112.18				
SkBF 2nd finger immersed hand (PU)	Cold	PAR	39.76±40.58	53.63±26.98	86.18±53.62	15.88±10.68	0.52	0.74	0.40	0.39
		AB	87.25±123.03	111.69±107.21	109.70±63.59	12.85±2.27				
	Neutral	PAR	172.11±102.98	162.28±69.79	106.03±73.03	239.5±287.83	0.26	0.59	0.06	0.96
		AB	201.79±128.36	222.09±125.80	110.11±63.12	42.04±43.51				
	Hot	PAR	374.34±83.81	373.12±61.66	193.86±89.52	179.29±104.79	0.71	0.91	0.14	0.01
		AB	321.51±64.45	302.48±90.80	182.64±64.84	178.57±117.44				
SkBF 2nd finger non immersed hand (PU)	Cold	PAR	25.62±20.04	14.01±6.08	15.51±4.59	10.56±1.61	0.45	0.56	0.43	0.70
		AB	44.37±55.95	39.63±64.00	18.71±9.46	14.79±8.45				
	Neutral	PAR	118.70±92.92	100.84±82.34	64.55±61.81	47.85±58.15	0.01	0.30	0.62	0.39
		AB	118.02±57.27	79.85±57.43	35.38±23.71	30.45±25.13				
	Hot	PAR	328.34±78.41[0.004]	309.00±104.31	216.83±130.70	216.54±167.29	1.22	0.42	0.14	0.13
		AB	233.74±77.01	273.39±60.96	230.44±41.23	231.42±12.43				
Sweat rate forehead ($mg/cm^2/min$)	Cold	PAR	1.27±0.40	1.40±0.36	1.53±0.39	1.76±0.48	0.14	0.20	0.12	0.45
		AB	1.22±0.33	1.46±0.22	1.57±0.32	1.51±0.60				
	Neutral	PAR	1.17±0.50	1.32±0.48	1.40±0.57	1.49±0.17	0.56	0.46	0.39	2.89
		AB	0.96±0.20	1.14±0.28	1.21±0.39	0.74±0.33				
	Hot	PAR	1.51±0.44	1.73±0.53	1.86±0.62	1.71±0.73	0.04	0.05	0.01	0.15
		AB	1.49±0.71	1.70±0.89	1.85±0.94	1.55±1.22				
Sweat rate leg ($mg/cm^2/min$)	Cold	PAR	0.72±0.54	0.70±0.53	0.68±0.55	0.88±0.67	0.06	0.06	0.17	0.13
		AB	0.69±0.40	0.67±0.46	0.77±0.43	0.80±0.63				
	Neutral	PAR	0.60±0.28	0.69±0.47	0.68±0.46	0.98±0.62	0.11	0.08	0.05	0.83
		AB	0.64±0.34	0.73±0.47	0.66±0.37	0.52±0.47				
	Hot	PAR	0.77±0.67	0.91±0.92	1.29±0.80	1.88±0.92	1.24	0.77	0.39	1.22
		AB	1.41±0.28	1.46±0.43	1.59±0.73	1.01±0.41				
Heart rate (beats/min)	Cold	PAR	71.29±13.76	71.86±16.20	69.77±14.78	70.27±15.58	0.40	0.47	0.06	0.18
		AB	66.76±7.89	66.03±7.18	70.39±6.01	68.21±4.84				
	Neutral	PAR	73.69±14.07	74.45±15.54	71.72±11.46	67.14±11.46	0.58	0.71	0.41	0.00
		AB	67.24±6.83	66.26±4.94	68.25±3.55	67.16±5.67				
	Hot	PAR	80.31±3.41	78.87±3.18	75.12±5.48	66.86±2.84	1.02	1.00	0.36	0.31

Table S2. Physiological and perceptual parameters in paraplegic (PAR) and able-bodied (AB) individuals in the tested environments. Effect sizes indicate between group comparisons.

			Baseline	Warm immersion	Cold immersion	Recovery	Effect size (d)			
							Base	WI	CI	Rec
		AB	74.16±7.80	73.89±6.30	72.96±6.58	67.95±4.14				
Systolic blood press. (mmHg)	Cold	PAR	122.48±20.74	116.86±19.75	127.54±22.70	108.00±3.61	0.92	0.54	0.42	0.36
		AB	107.93±8.61	108.86±6.79	120.24±9.22	113.67±21.73				
	Neutral	PAR	113.86±19.67	113.17±19.20	121.83±19.38	109.00±7.07	0.61	0.42	0.15	1.38
		AB	104.78±7.52	106.33±12.68	124.44±16.20	122.00±11.31				
	Hot	PAR	108.33±13.91	110.17±22.71	117.50±17.02	98.50±4.95	0.41	0.41	0.42	0.28
		AB	103.39±10.03	103.00±10.49	111.40±11.75	97.50±0.71				
Diastolic blood press. (mmHg)	Cold	PAR	70.86±8.48	73.86±10.87	83.18±11.47	76.33±18.58	0.30	0.22	0.41	0.41
		AB	73.21±7.44	72.00±4.28	87.05±7.06	83.00±13.86				
	Neutral	PAR	69.20±13.01	73.50±13.84	79.77±14.47	65.50±3.54	0.28	0.22	0.69	3.43
		AB	72.06±6.86	71.00±8.44	87.88±8.32	75.50±2.12				
	Hot	PAR	67.36±11.45	68.67±17.74	73.61±13.28	66.00±8.49	0.42	0.05	0.45	1.06
		AB	71.11±5.30	68.00±6.07	78.22±5.53	75.00±8.49				
Pain index hand (likert)	Cold	PAR	-	-	5.98±2.27	-	-	-	0.34	-
		AB	-	-	6.65±1.60	-				
	Neutral	PAR	-	-	4.50±2.33	-	-	-	0.91	-
		AB	-	-	6.43±1.85	-				
	Hot	PAR	-	-	2.79±0.81	-	-	-	1.24	-
		AB	-	-	4.90±2.27	-				
Pain index hand (VAS)	Cold	PAR	-	-	5.49±2.28	-	-	-	0.45	-
		AB	-	-	6.43±1.84	-				
	Neutral	PAR	-	-	3.40±2.21	-	-	-	1.25	-
		AB	-	-	6.01±1.96	-				
	Hot	PAR	-	-	2.90±1.31	-	-	-	0.80	-
		AB	-	-	4.46±2.45	-				
Distress index hand (likert)	Cold	PAR	-	-	6.02±2.28	-	-	-	0.43	-
		AB	-	-	6.88±1.63	-				
	Neutral	PAR	-	-	4.72±2.61	-	-	-	0.73	-
		AB	-	-	6.41±2.01	-				
	Hot	PAR	-	-	2.76±0.82	-	-	-	1.37	-
		AB	-	-	4.99±2.16	-				
Tactile sensitivity (monofil.)	Cold	PAR	0.38±0.79	0.35±0.73	0.88±1.29	-	0.01	0.00	0.44	-
		AB	0.39±0.79	0.35±0.73	1.31±0.43	-				
	Neutral	PAR	0.14±0.15	0.07±0.00	25.74±60.98	-	0.63	0.58	0.49	-
		AB	0.07±0.00	0.13±0.13	4.48±8.03	-				
	Hot	PAR	0.45±0.87	0.39±0.79	1.12±0.76	-	0.00	0.58	0.56	-
		AB	0.46±0.86	0.07±0.00	25.40±61.07	-				
Tactile sensitivity (esthesiom.)	Cold	PAR	2.92±0.96	2.29±1.65	2.57±1.27	-	0.47	0.93	0.88	-
		AB	2.52±0.78	3.51±0.88	4.12±2.14	-				
	Neutral	PAR	2.49±1.00	2.88±0.89	2.93±1.41	-	0.39	0.08	0.15	-
		AB	2.87±0.98	2.95±0.86	3.11±1.04	-				
	Hot	PAR	1.70±1.19	2.01±1.39	2.37±1.27	-	1.00	0.33	0.68	-
		AB	2.70±0.78	2.38±0.79	3.03±0.54	-				
Thermal comfort	Cold	PAR	2.33±1.03	2.43±1.13	2.56±0.62	2.17±0.29	0.38	0.42	0.26	0.35
		AB	2.00±0.68	2.00±0.87	2.72±0.60	2.50±1.32				
	Neutral	PAR	1.31±0.43	1.33±0.52	1.31±0.29	1.33±0.29	0.24	0.64	0.48	0.65
		AB	1.22±0.27	1.08±0.20	1.50±0.47	1.83±1.04				
	Hot	PAR	1.58±0.67	1.83±1.21	1.70±1.07	1.50±0.00	0.32	0.29	0.19	1.00
		AB	1.78±0.53	2.17±1.08	1.53±0.58	1.25±0.35				
Thermal sensation	Cold	PAR	-1.95±0.62	-1.86±0.69	-2.16±0.63	-0.67±2.31	0.72	1.03	0.41	0.91
		AB	-1.52±0.57	-1.14±0.69	-1.90±0.64	-2.33±1.15				
	Neutral	PAR	-0.22±1.43	-0.17±1.33	-0.11±1.19	0.00±2.00	0.16	0.00	0.23	0.82
		AB	-0.39±0.44	-0.17±0.98	-0.33±0.54	-1.33±1.15				
	Hot	PAR	1.50±1.07	1.67±0.82	1.60±0.86	1.50±0.71	0.13	0.00	0.78	0.00
		AB	1.61±0.49	1.67±0.82	0.97±0.74	1.50±0.71				

Table S2. Physiological and perceptual parameters in paraplegic (PAR) and able-bodied (AB) individuals in the tested environments. Effect sizes indicate between group comparisons.

	Baseline	Warm immersion	Cold immersion	Recovery	Effect size (d)			
					Base	WI	CI	Rec

Key: Superscript numbers indicate p values for statistically significant differences at p<0.008 from paired samples t tests with a Bonferroni correction.

Note: Base = baseline; WI = warm immersion; CI = cold immersion; Rec = recovery; SkBF = skin blood flow; VAS = visual analogue scale.

	very small	small	medium	large	very large	huge
**Effect size (d) interpretation:**	0.01-0.19	0.20-0.49	0.50-0.79	0.80-1.19	1.20-1.99	≥2.0
higher values in PAR group						
lower values in PAR group						
